# EGFR-dependent tyrosine phosphorylation of integrin β4 is not required for downstream signaling events in cancer cell lines

**DOI:** 10.1038/s41598-021-88134-6

**Published:** 2021-04-21

**Authors:** Lisa te Molder, Maaike Kreft, Niels Heemskerk, Joyce Schuring, Jose M. de Pereda, Kevin Wilhelmsen, Arnoud Sonnenberg

**Affiliations:** 1 Division of Cell Biology I, The Netherlands Cancer Institute, Plesmanlaan 121, 1066 CX Amsterdam, The Netherlands; 2 Centro de Investigación del Cáncer and Instituto de Biología Molecular y Celular del Cáncer, Consejo Superior de Investigaciones Científicas (CSIC), Universidad de Salamanca, 37007 Salamanca, Spain; 3 Present Address: Department of Molecular Cell Biology and Immunology, Cancer Center Amsterdam, Amsterdam UMC, Vrije Universiteit Amsterdam, De Boelelaan 1117, Amsterdam, The Netherlands; 4 Present Address: Bioceros, Yalelaan 46, 3584 CM Utrecht, The Netherlands; 5 Present Address: BioAge Labs, 1445A S 50th St, Richmond, CA 94804 USA

**Keywords:** Proteins, Integrins, Biochemistry, Cell biology, Molecular biology

## Abstract

In epithelial cancers, the epidermal growth factor receptor (EGFR) and integrin α6β4 are frequently overexpressed and found to synergistically activate intracellular signaling pathways that promote cell proliferation and migration. In cancer cells, the β4 subunit is phosphorylated at tyrosine residues not normally recognized as kinase substrates; however, the function of these phosphotyrosine residues in cancer cells is a subject of much debate. In EGFR-overexpressing carcinoma cells, we found that the Src family kinase (SFK) inhibitor PP2 reduces β4 tyrosine phosphorylation following the activation of EGFR. However, siRNA mediated knockdown of the SFKs Src, Fyn, Yes and Lyn, individually or in combination, did not affect the EGF-induced phosphorylation of β4. Using phospho-peptide affinity chromatography and mass spectrometry, we found that PLCγ1 binds β4 at the phosphorylated residues Y1422/Y1440, but were unable to verify this interaction in A431 carcinoma cells that overexpress the EGFR. Furthermore, using A431 cells devoid of β4 or reconstituted with phenylalanine specific mutants of β4, the activation of several downstream signaling pathways, including PLCγ/PKC, MAPK and PI3K/Akt, were not substantially affected. We conclude that tyrosine-phosphorylated β4 does not enhance EGFR-mediated signaling in EGFR-overexpressing cells, despite the fact that this integrin subunit is highly tyrosine phosphorylated in these cells.

## Introduction

Cellular behavior is regulated by a multitude of intracellular signaling pathways. These signaling pathways, which are activated by environmental cues such as hormones or growth factors (GFs), regulate proliferation, survival and migration of the cells^1^. In tumor cells, normal signaling is frequently impaired due to increased expression of or mutations in receptor-tyrosine kinases (RTKs), such as the epidermal growth factor receptor (EGFR) (reviewed in Ref.^2^). Additionally, the expression of integrins, which are cell surface receptors that mediate interaction between cells and the extracellular matrix, is frequently deregulated in tumor cells (reviewed in Ref.^3^).

Integrin α6β4, which is a receptor for laminin, is expressed by a variety of epithelial tissues and cell types^4^. In stratified and pseudostratified epithelia, the integrin is a major component of hemidesmosomes^5^, in which it mediates anchorage of cytoskeletal intermediate filaments to the plasma membrane through interaction with the plakin family members BP230 and plectin^6,7^. Overexpression and mislocalization of this integrin has been correlated with cancer progression and poor prognosis of multiple tumor types (reviewed in Refs.^8–10^). Evidence suggests that integrin α6β4 contributes to cancer progression and cell migration by promoting PI3K/Akt and Ras/MAPK signaling in cells^11–18^. However, how β4 contributes to the activation of intracellular signaling pathways is still a point of discussion. Upon binding to its ligand, clustering of β4 and cross-phosphorylation of β4 by non-receptor tyrosine kinases has been shown to contribute to downstream signaling in carcinomas^19^. Alternatively, part of β4’s contribution to signaling could occur upon RTK activation, such as the EGFR^20,21^.

The EGFR is a RTK that is activated by binding of its ligand EGF. It activates multiple signaling pathways, including the PI3K/Akt and MAPK signaling pathways^22^. Furthermore, in some cell types, EGF also activates STAT3 and PLCγ/PKC signaling pathways^23–25^. Some of the kinases activated downstream of the EGFR, are involved in the phosphorylation of residues in the cytoplasmic domain of β4. The bulk of these residues reside in the connecting segment (CS) between the two pairs of fibronectin type III (FnIII) domains (FnIII-1,2 and FnIII-3,4), although some sites in FnIII-3,4 and in the C-terminal tail of β4 have been reported to be phosphorylated as well^26^.

Phosphorylation of β4 on serines 1356, 1360, 1364 and 1424 in the CS, and threonine 1736 in the C-tail promotes HD disassembly by interfering with β4-plectin and β4-BP180/BP230 binding^27–31^. On the contrary, phosphorylation of β4 on tyrosines,Y1422 and Y1440 in the CS, Y1257 in FnIII-2 and Y1494, Y1526 and Y1642 in FnIII-3,4, has been suggested to play a role in the activation of the PI3K/Akt and MAPK signaling pathways, via binding and activation of adaptor proteins, like Shc and IRS1^10,11,18,21, 32–34^. However, the importance of β4 tyrosine phosphorylation for cancer progression is unclear which is primarily due to the use of various cell types and assays (2D, 3D or in vivo)^35^.

In this paper we investigated the crosstalk between integrin β4 and the EGFR in the activation of cancer promoting signaling pathways in a variety of cell lines. Special attention was paid to the role of the tyrosine residues in the β4 CS, since their role in growth factor-stimulated signaling pathways has remained controversial. Our studies confirm previous findings that β4 is tyrosine phosphorylated in A431 cells overexpressing the EGFR but excluded a role of Src family kinases (SFKs) in the EGFR-stimulated phosphorylation of β4. Furthermore, we demonstrate that the phosphorylated residues Y1422 and 1440 in β4 can function as binding sites for PLCγ1 in vitro, but that they do not contribute substantially to signaling from the EGFR to the PLCy1/PKC, PI3K/Akt and MAPK pathways in cells.

## Results

### EGF-mediated tyrosine phosphorylation of β4 is correlated with EGFR overexpression

**Figure 1:**
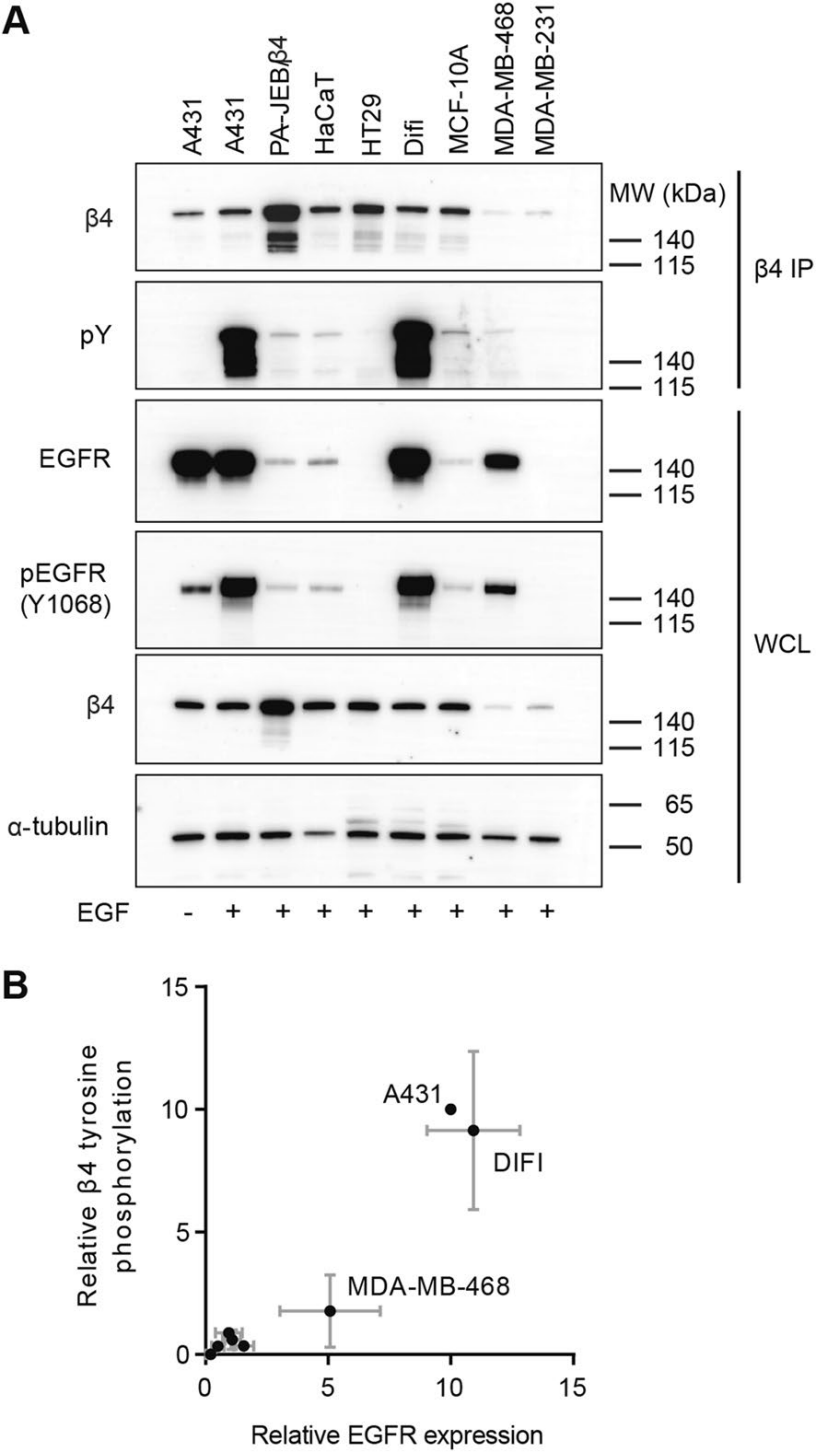
EGF-mediated tyrosine phosphorylation of β4 occurs in EGFR overexpressing cell lines. (**A**) whole cell lysates (WCL) and β4 immunoprecipitation (IP) samples of multiple transformed and untransformed cell lines, expressing β4 and EGFR at different levels, treated with EGF for 5 min after 20 h of serum starvation, were analyzed by Western blot for total β4 levels, tyrosine phosphorylation of β4, total EGFR levels, tyrosine phosphorylation of EGFR and α-tubulin levels (loading control). (**B**) The protein and tyrosine abundances were quantified, normalized to the levels of A431 (at 10), and visualized in a graph. β4 tyrosine phosphorylation (IP) is normalized to total β4 levels (IP) on the Y-axis, and total EGFR levels (WCL) were normalized to α-tubulin levels (WCL) on the X-axis. The graph shows the mean + standard deviation (SD) of 3 independent experiments. Uncropped images of merged chemiluminescent and colorimetric blots obtained with a ChemiDoc imaging system (BioRad) are shown in Suppl. Fig. 1.

To investigate possible crosstalk of integrin β4 with the EGFR in the promotion of cancer signaling, we first characterized EGFR-mediated β4 tyrosine phosphorylation in multiple benign and malignant epithelial cell lines derived from breast, colon and skin tissues. By analyzing whole cell lysates of the different cell lines by Western blot, we observed that the total β4 and EGFR expression levels varied strongly among the different cell lines. Furthermore, the expression levels of β4 and EGFR do not appear to be related to each other or to their tissue of origin. However, we did observe a clear relationship between tyrosine phosphorylation of β4 and the levels of EGFR in cells treated with EGF (Fig. 1A). This effect was independent of the tissue of origin, since the two cell lines which had the highest levels of EGFR expression and β4 tyrosine phosphorylation, Difi and A431, were derived from colon and skin tissue, respectively (Fig. 1A,B). Furthermore, these assays showed that β4 tyrosine phosphorylation occurred specifically in the presence of EGF, since tyrosine phosphorylation of β4 was not observed in serum-starved cells (Fig. 1A). In conclusion, the amount of β4 that is tyrosine phosphorylated is independent of tissue origin and the total amount of β4 in the cells, but is dependent on the presence of EGF and correlated with the amount of EGFR in the cells.

### β4 tyrosine phosphorylation occurs abundantly on the β4 connecting segment

**Figure 2:**
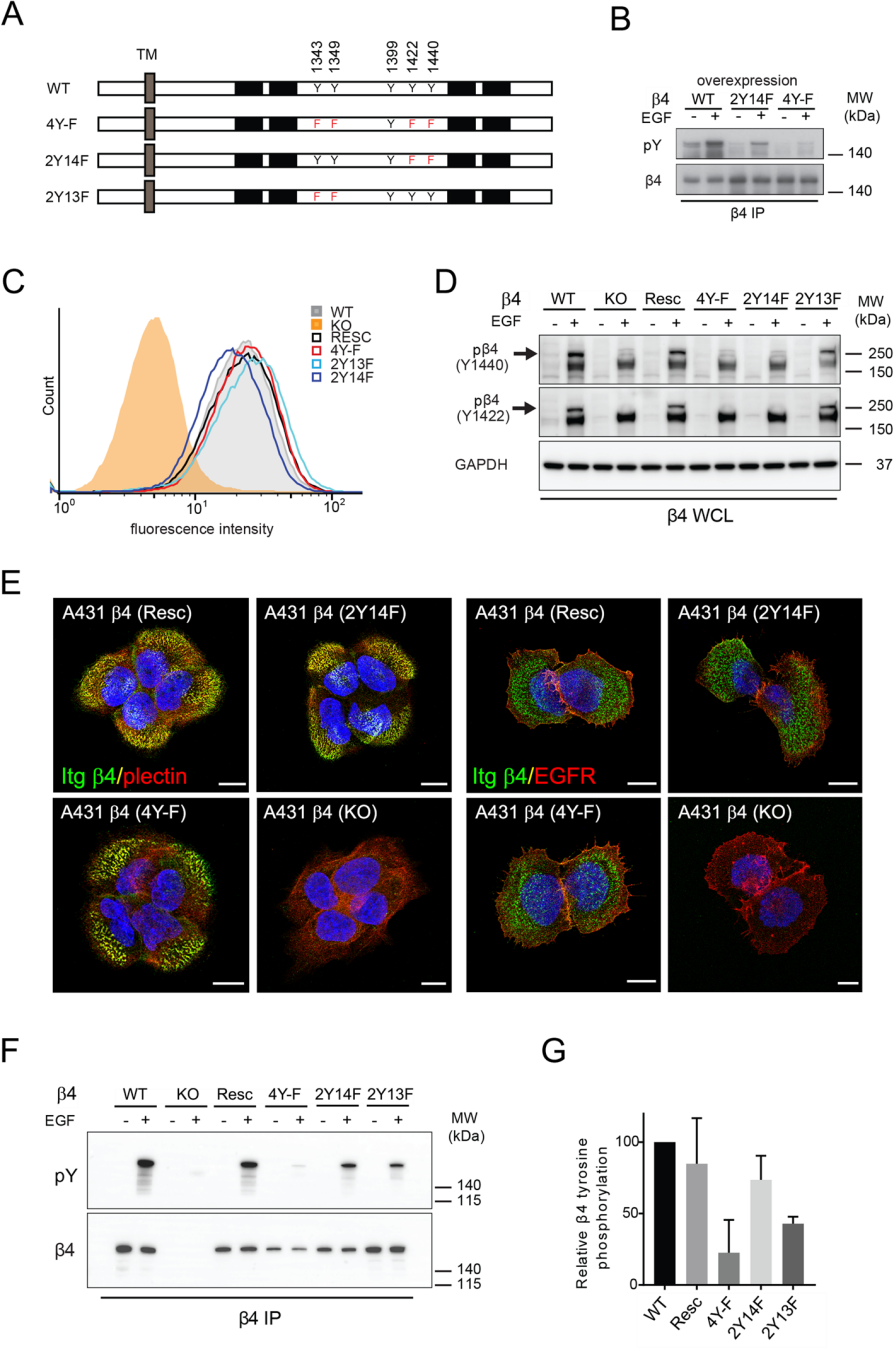
β4 phosphorylation occurs abundantly on the β4 CS. (**A**) Representation of the tyrosines (Y) present and mutated to phenylalanines (F) in the connecting segment (CS) of the various β4 mutants (4Y-F, 2Y14F and 2Y13F) and wild type (WT) β4. (**B**) EGF-induced β4 tyrosine phosphorylation of β4 tyrosine mutants shown by Western blot analyses of β4 immunoprecipitation (IP) samples of EGF-treated and untreated COS7 cell overexpressing WT or mutant β4 together with the EGFR. Blots were probed for β4 and phospho-tyrosine (pY). (**C**) Comparable β4 surface levels in A431 cell lines analyzed by FACS. The A431 cell lines analyzed are as follows: WT, expressing endogenous β4; KO, depleted of endogenous β4 by CRISPR-Cas9; Resc, β4 (KO) cells reconstituted with WT β4; 4Y-F, 2Y13F and 2Y14F β4 mutants, β4 (KO) cells reconstituted with mutated β4. (**D**) Representative Western blot analyses of WCLs of the various A431 cells for phosphorylation of β4 at Y1440 and Y1422 using phosphosite-specific antibodies. Cells were untreated or treated with EGF for 5 min after 20 h serum starvation. (**E**) Double immunofluorescence staining for β4 and plectin, and β4 and the EGFR in A431 β4 (KO) cells and β4 (KO) cells reconstituted with WT β4, 4Y-F or 2YF14 β4. Scale bars: 10 µm. (**F**) Representative Western blot analyses of β4 tyrosine phosphorylation and total β4 levels in β4 IP samples obtained from the various A431 cells untreated or treated with EGF for 5 min after 20 h serum starvation. (**G**) Quantification of β4 tyrosine phosphorylation levels in the various A431 cell lines, per sample normalized to total β4 levels and per experiment to the pY/β4 WT levels. Graphs shows the mean + SD from 3 independent experiments. Uncropped images of western blots, and merged chemiluminescent and colorimetric blots obtained with a ChemiDoc imaging system (BioRad) are shown in Suppl. Fig. 2.

Multiple tyrosine phosphorylation sites have been identified in the CS of β4^26^. To study which of these sites are phosphorylated downstream of EGFR activation and whether they play a role in β4 signaling in conjunction with the EGFR, we generated constructs in which specific tyrosines in the β4 CS were mutated to phenylalanines (Fig. 2A). Firstly, the 2Y14F and the 4Y-F β4 mutants were transiently expressed together with the EGFR in COS7 cells and β4 tyrosine phosphorylation induced by EGF was analyzed (Fig. 2B). In the 2Y14F mutant, in which two well-studied tyrosine residues, Y1422 and Y1440, were mutated, tyrosine phosphorylation of β4 was clearly reduced. But in the 4Y-F mutant in which Y1343 and Y1349 were mutated in addition to Y1422 and Y1440, β4 tyrosine phosphorylation was almost undetectable (Fig. 2B). This suggests that in addition to Y1422 and Y1440, these other CS tyrosines are phosphorylated upon EGFR activation. The Y1343 and Y1349 sites were identified as tyrosine phosphorylation sites on β4 in several mass spectrometry data sets^26^, but have not yet been studied using cell biological assays.

To examine the extent and contribution of the different tyrosine phosphorylation sites in the CS of β4 at endogenous levels, we depleted β4 in A431 cells using CRISPR-Cas9 and reintroduced wild-type β4 or the different β4 tyrosine mutants, including the 2Y13F mutant in which only the Y1343 and Y1349 have been mutated, in these knockout cells. FACS analysis showed that the surface levels of β4 in the stably transformed cells expressing wild-type or mutant β4 proteins were comparable to one another and to the endogenous surface levels of β4 on A431 cells (Fig. 2C). Furthermore, using phospho-site specific antibodies, we confirmed the absence of tyrosine phosphorylation on Y1440 and Y1422 in the 2Y14F and 4Y-F mutants, but not in the 2Y13F mutant (Fig. 2D). All β4 mutants co-localized with plectin in hemidesmosomal-like adhesion structures similarly to wild type β4. By contrast, β4 did not obviously co-localize with the EGFR at these adhesion structures, although the two proteins were found in close proximity of each other (Fig. 2E). After treatment of the stably transformed cell lines with EGF, the levels of total tyrosine phosphorylation of β4 were assessed by Western blot with anti-phosphotyrosine antibodies (Fig. 2F). Quantification of the relative amount of β4 tyrosine phosphorylation confirmed that mutation of all four tyrosines drastically reduced the total amount of tyrosine phosphorylation of β4 and that in addition to Y1422 and Y1440, the Y1343 and Y1349 sites also contributed to β4 tyrosine phosphorylation (Fig. 2F,G). However, the data also shows that some residual tyrosine phosphorylation of β4 remains in the 4Y-F mutant, which suggests that additional sites such as Y1399, the only tyrosine left untouched in the β4-CS, are phosphorylated as well.

### Src family kinases do not contribute to β4 tyrosine phosphorylation

**Figure 3:**
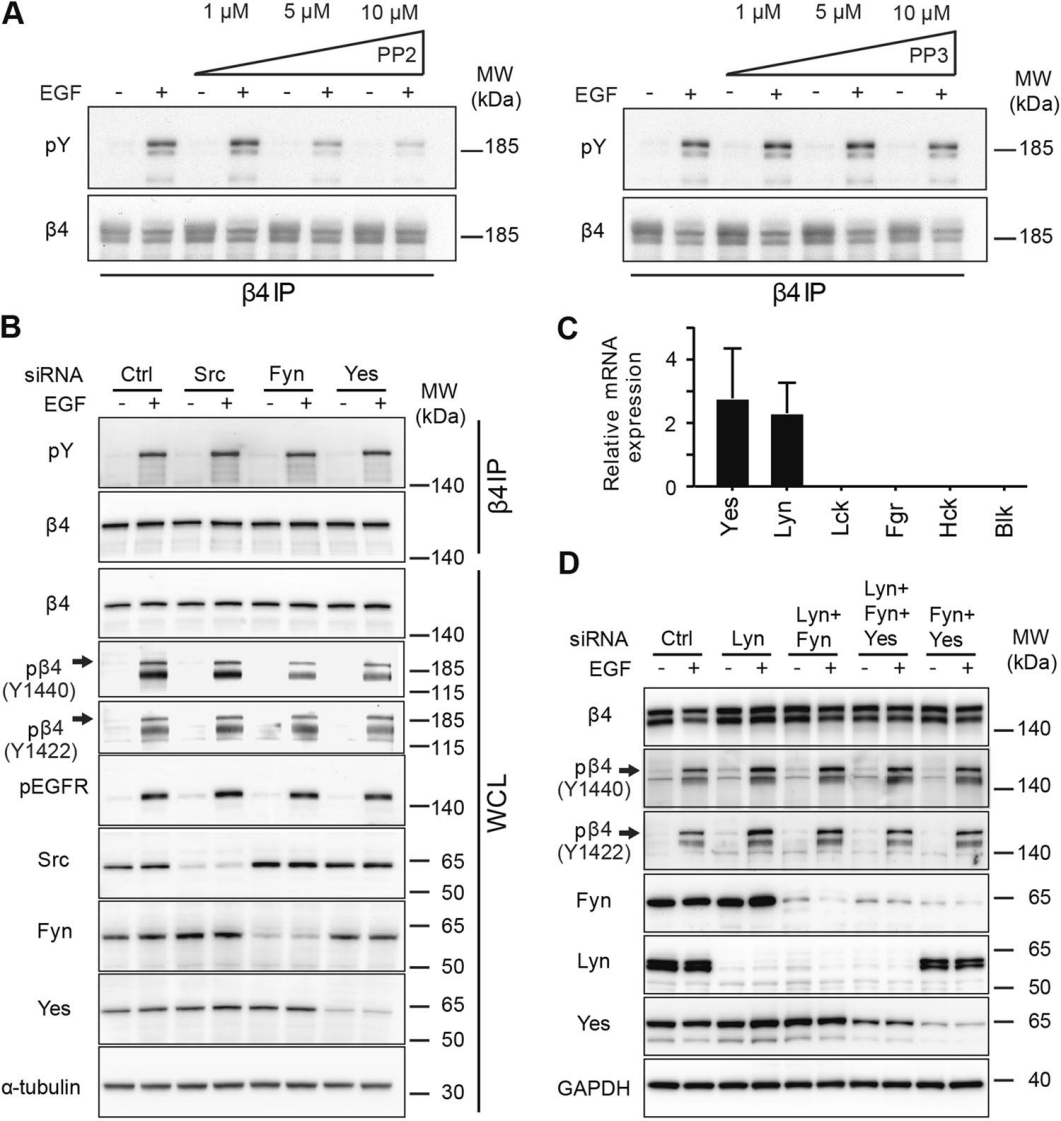
Effects of PP2 and siRNA-mediated knockdown of SFKs on the tyrosine phosphorylation of β4. (**A**) Western blot analyses of β4 tyrosine phosphorylation and total β4 levels in β4 immunoprecipitation (IP) samples of A431-β4-GFP (expressing both endogenous β4 and β4-GFP) cells untreated or treated for 5 min with EGF, after treatment with 0, 1, 5 or 10 µM PP2 or PP3. (**B**) Western blot analyses of β4 IP and WCL samples of WT A431 cells transfected with siRNAs for Src, Fyn and Yes and Ctrl siRNAs, untreated or treated for 5 min with EGF. The blots were proped for phospho-tyrosine (pY), total β4, pβ4 (Y1440), pβ4 (Y1422), pEGFR (Y1068), Src, Fyn, Yes and α-tubulin (loading control). (**C**) Relative mRNA expression of Yes, Lyn, Frk, Lck, Fgr, Hck and Blk SFKs in WT A431 cells analyzed by RT-PCR. Graph shows the mean + SD of 3 independent experiments. (**D**) Western blot analyses of WCL samples of WT A431 cells transfected with siRNAs for Fyn, Lyn and/or Yes or Ctrl siRNAs, untreated or treated for 5 min with EGF. The blots were probed for β4, pβ4 (Y1440), pβ4 (Y1422), Fyn, Yes, Lyn and GAPDH (loading control). Uncropped images of merged chemiluminescent and colorimetric blots obtained with a ChemiDoc imaging system (BioRad) and the repetition of the experiment in (**A**) are shown in Suppl. Fig. 3.

To examine which tyrosine kinase phosphorylates β4 upon EGFR activation in A431 cells, we focused our investigations on the Src family kinases (SFKs), which previously have been shown to be involved in tyrosine phosphorylation of β4^21,36^. Treatment of A431 cells with the SFK inhibitor PP2 showed a concentration dependent inhibition of EGF-induced tyrosine phosphorylation of β4. Such a reduction was not observed with the PP3 control (Fig. 3A). Since the PP2 inhibitor inhibits multiple SFKs, we performed siRNA-mediated knock-down of Src, Fyn and Yes, the most common SFKs^37^, and tested whether their knock-down has an effect on β4 tyrosine phosphorylation. Western blot analysis showed a strong reduction of Src, Fyn or Yes after transfection with their respective siRNAs, but total tyrosine phosphorylation of β4 was not reduced. Furthermore, although a small reduction in the phosphorylation of Y1422 and Y1440 was observed in the absence of Fyn or Yes (Fig. 3B), a decrease in the phosphorylation of these residues was not observed when expression of both Fyn and Yes were simultaneously reduced in the same cell (Fig. 3D). Expression analysis by quantitative RT-PCR showed that, in addition to Src, Fyn and Yes, A431 cells express the SFK Lyn but not Lck, Fgr, Hck and Blk (Fig. 3C). A common feature of α6β4, EGFR and the SFKs Fyn, Yes and Lyn is that these proteins are all palmitoylated and thus could be together in cholesterol- and sphingolipid rich, liquid ordered membrane domains^38–42^. Therefore, it was of interest to also study the contribution of Lyn to β4 tyrosine phosphorylation. SiRNA-mediated silencing of Lyn alone or in combination with Fyn, or Fyn and Yes, however, did not reduce the phosphorylation on β4 at Y1422 or Y1440 (Fig. 3D). In conclusion, our data show that although the PP2 inhibitor reduces tyrosine phosphorylation of β4 upon EGFR activation, Src and the palmitoylated SFKs, Fyn, Yes and Lyn are not essential for tyrosine phosphorylation of β4.

### β4 Y1422 and Y1440 provide a docking platform for PLCγ1

**Figure 4:**
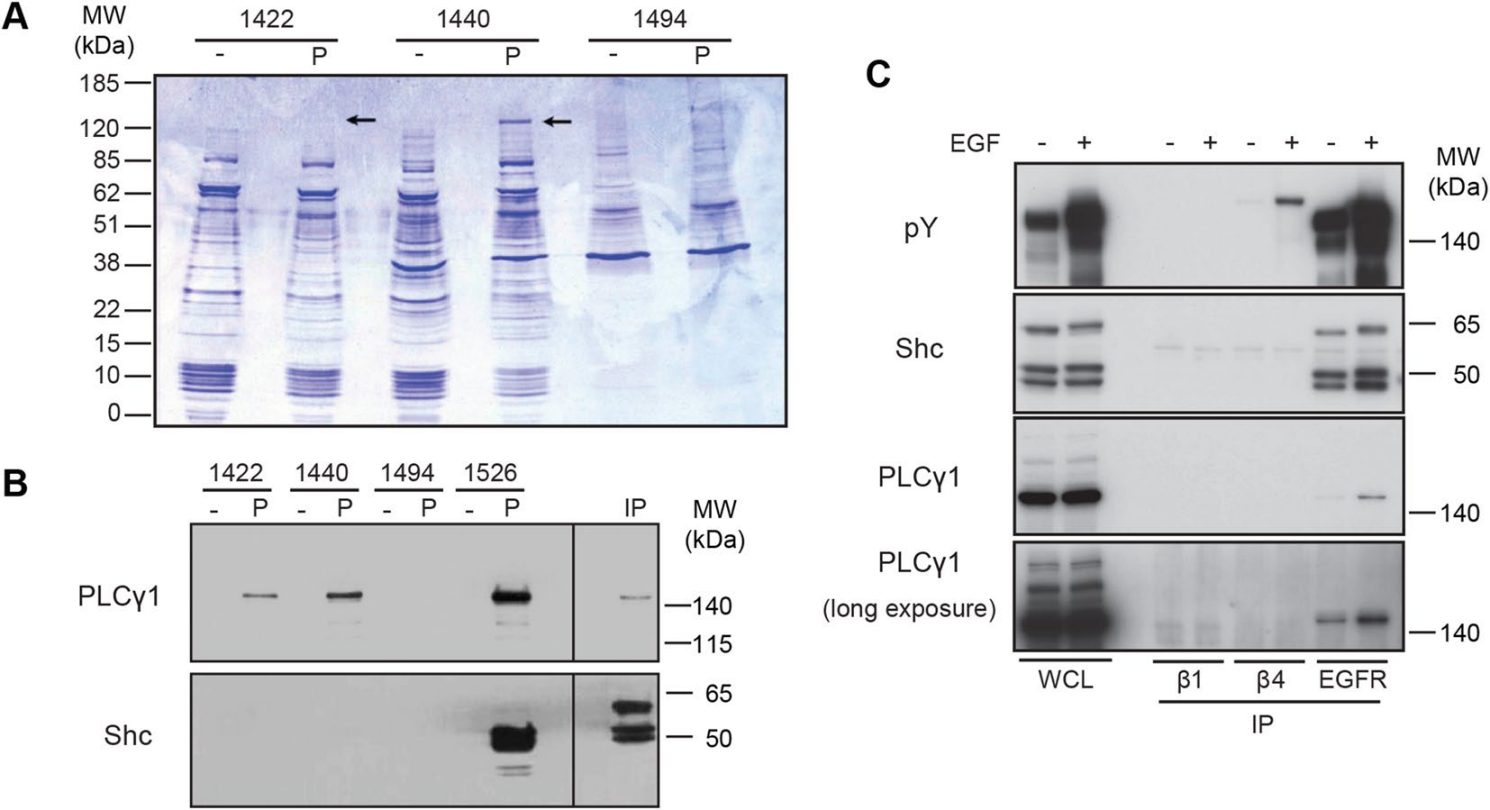
β4 tyrosine phosphorylation might provide a docking platform for PLCγ1. (**A**) A431 cells were lysed and different synthetic phosphorylated (P) and unphosphorylated (–) peptides derived from β4 were used for immunoprecipitation (IP). IPs were separated on gel and stained with Coomassie blue. The arrows indicate the protein band identified by mass spectrometry as PLCγ1. (**B**) A431 cells were lysed and different phosphorylated (P) and unphosphorylated (–) synthetic peptides derived from β4 were used for IP. IP samples were analyzed by Western blot for Shc and PLCγ1. (**C**) A431 cells were serum starved and subsequently treated with EGF for 5 min. A431 WCL and β1, β4 and EGFR IP samples were analyzed by WB for phospho tyrosine, Shc and PLCγ1. Molecular weight (MW) markers are indicated. Uncropped images of merged chemiluminescent and colorimetric blots obtained with a ChemiDoc imaging system (BioRad) are shown in Suppl. Fig. 4.

In order for β4 to use tyrosine phosphorylation for intracellular signaling, it must bind proteins that contain a phospho-tyrosine binding domain, such as an SH2 or PTB domain. The Y1422 and Y1440 sites have been reported to bind the intracellular signaling adapter protein Shc^16,43^. To investigate the interactions of the phosphorylated Y1422 and Y1440 residues in keratinocytes, we performed peptide pull down experiments from lysates of PA-JEB/β4 keratinocytes with phosphorylated and unphosphorylated peptides containing Y1422, Y1440, or Y1494, a non-CS-tyrosine residue which previously has been shown to regulate multiple signaling pathways important for tumor development and progression^18,32,44,45^. By SDS gel electrophoresis, we observed a prominent 135 kDa protein in the pulldowns with phosphorylated Y1440 (pY1440). A band of similar molecular weight is also observed in the pulldown with the phosphorylated Y1422 (pY1422) peptide, but not with phosphorylated Y1494 (pY1494) or the corresponding unphosphorylated peptides (Fig. 4A). Mass spectrophotometry analysis identified this band as PLCγ1.

PLCγ1 contains two tandem SH2 domains, which likely are responsible for the binding of this phospholipase to the two phosphorylated tyrosine residues in β4. By analyzing similar pulldown experiments using Western blot, we confirmed the interaction of PLCγ1, but not Shc, with pY1422 and pY1440 (Fig. 4B). Additionally, we showed that both Shc and PLCγ1 bind the phosphorylated peptide Y1526 (pY1526), which resides in FnIII-3 of β4. However, the crystal structure of the FnIII-3 domain revealed that the structural environment of Y1526 is incompatible with binding SH2 or PTB domains^46^. Hence, it is not likely that either Shc or PLCγ1 can bind this phosphorylated tyrosine residue in the context of a competently folded FnIII-3 domain. To further study the interaction of PLCγ1 (and Shc) with integrin β4 under more physiologically relevant conditions, we used untreated and EGF-treated A431 cells and performed β4 immunoprecipitations (Fig. 4C). Under these conditions, as well as in cells exogenously overexpressing integrin β4 (unpublished data), we were unable to confirm its interaction with either Shc or PLCγ1. In contrast, we could confirm the interaction of Shc and PLCγ1 with the phosphorylated EGFR.

In conclusion, we show that phospho-peptides based on the β4 sequence containing residues Y1440 and Y1422 associate with PLCγ1, but not Shc, in pull down assays. However, we were unable to detect an association between PLCγ1 and β4 in A431 cells.

### β4 and its tyrosine phosphorylation does not alter downstream signaling of the EGFR

**Figure 5:**
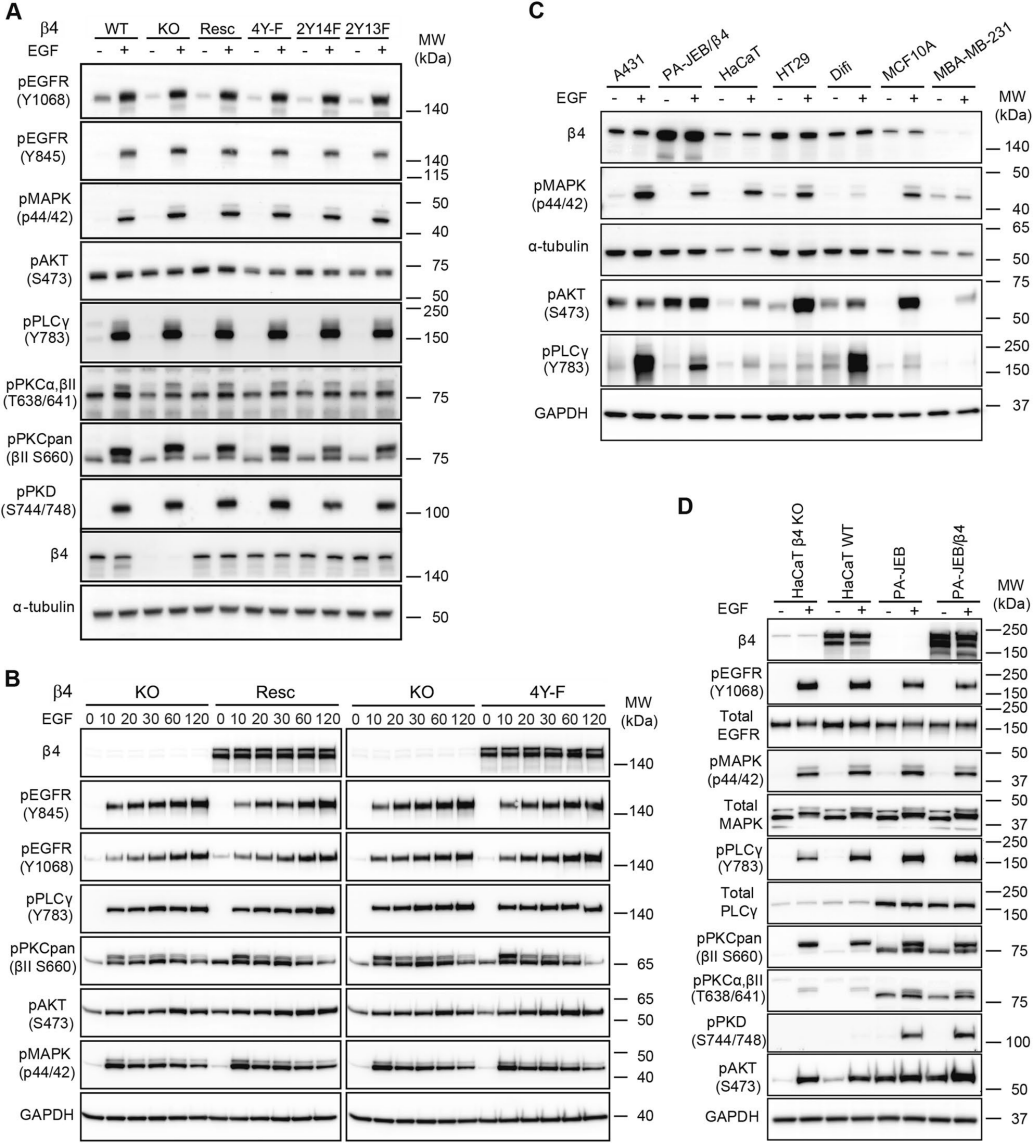
β4 and its tyrosine phosphorylation at the CS does not alter signaling downstream of the EGFR. (**A**) Western blot analyses of WCLs from various WT and β4 mutant A431 cell lines, untreated or treated for 5 min with EGF after 20 h serum starvation, for pEGFR (Y845 and Y1068), pMAPK, pAKT, pPLCγ1, pPKCα, βII (T638/641), pPKCpan (βII S660), pPKD (S744/748), β4 and α-tubulin (loading control). (**B**) Western blot analyses of A431 β4 (Resc), β4 (KO) and β4 (4Y-F) cells untreated or treated for different timepoints (0, 10, 20, 30, 60 and 120 min) with EGF after 20 h serum starvation, for β4, pEGFR (Y845 and Y1068), pPLCγ1 (Y783), pPKCpan (βII S660), pAKT (S473), pMAPK (p44/42) and GAPDH (loading control). (**C**) Western blot analyses of A431, PA-JEB/β4, HaCaT, HT29, Difi, MCF10A, MDA-MB-231 cells, untreated and treated with EGF for 5 min after 20 h serum starvation, β4, pMAPK (p44/42), pAKT (S473), pPLCγ1 (Y783), α-tubulin and GAPDH (loading controls). (**D**) Western blot analyses of PA-JEB/β4 and HaCaT cells, β4 proficient and deficient, treated with or without EGF for 10 min after 20 h serum starvation, for total β4, pEGFR (Y1068), total EGFR, pMAPK (p44/42), total MAPK, pPLCγ1 (Y783), total pPLCγ1, pPKCpan (βII S660), pPKCα,β II (T638/641), pPKD (S744/748), pAKT(S473) and GAPDH (loading control). Of note, while phosphorylation of PLCγ1 appears reduced in HaCaT β4 KO cells, this reduction was not consistently observed in repeat experiments. Uncropped images of merged chemiluminescent and colorimetric blots obtained with a ChemiDoc imaging system (BioRad) are shown in Suppl. Fig. 5.

To investigate whether tyrosine phosphorylation of the β4-CS contributes to EGF-induced signal transduction, we compared the activation of signaling molecules in pathways downstream from the EGFR in β4 knockout (KO) A431 cells and A431-β4 (KO) cells reconstituted with wild-type (Resc) or mutant β4 proteins (4Y-F, 2F14Y and 2F13Y). By Western blot analysis, we observed no differences in the level of EGFR phosphorylation between β4 knockout cells and reconstituted cells, suggesting that β4 does not modulate EGFR kinase activity. ERK1/2 and Akt, two well-known downstream signaling molecules of the EGFR, were also equally activated among the different cell lines (Fig. 5A). The same results were observed when β4 proficient and deficient A431 cells were treated for different periods of time with EGF (Fig. 5B). Furthermore, although we identified PLCγ1 as a potential binding partner of tyrosine phosphorylated β4, PLCγ1 and PKC (downstream kinase of PLCγ1) activation downstream of the EGFR receptor were not dependent on tyrosine phosphorylation of β4 (Fig. 5A,B). These results show that neither β4 nor its tyrosine phosphorylation substantially affect EGFR activity or downstream signaling events in A431 cells.

To investigate whether β4 affects EGFR-mediated signaling pathways in other cell types, we first characterized the signaling pathways that are induced by EGF in multiple benign and malignant cell lines. All cell lines, except MDA-MB-231 cells and DiFi rectal carcinoma, responded robustly to EGF stimulation by phosphorylating ERK1/2 (Fig. 5C). In contrast, activation of the PI3K/Akt and PLCγ1 signaling pathways varied considerably among the cell lines. While some cell lines already had a high basal PI3K/Akt activity, which cannot be further induced by EGF stimulation, others displayed a clear increase in the levels of pAkt in response to EGF stimulation. Strong PLCγ1 phosphorylation was observed in EGF-stimulated A431 and Difi cells, which both overexpress the EGFR (Figs. 1A and 5C). To assess the contribution of β4 to EGFR signaling, we selected two non-cancerous cell lines PA-JEB/β4 and HaCat keratinocytes, which displayed only moderate levels of PLCγ1 phosphorylation upon EGF stimulation, but differed in the degree of constitutive phosphorylation of Akt. Comparison of the effects of β4 expression on the phosphorylation of PLCγ/PKC signaling pathway components, as well as those of the MAPK/ERK and PI3K/Akt signaling pathways, did not reveal any detectable difference in their phosphorylation status irrespective of EGF treatment (Fig. 5D).

In conclusion, we show that signaling downstream of the EGFR differs between cell lines, but β4 or its tyrosine phosphorylation does not affect the signaling of the EGFR, neither in cancer cells nor in non-cancerous epithelial cells.

### EGFR activation in A431 cells promotes growth-arrest rather than proliferation

**Figure 6:**
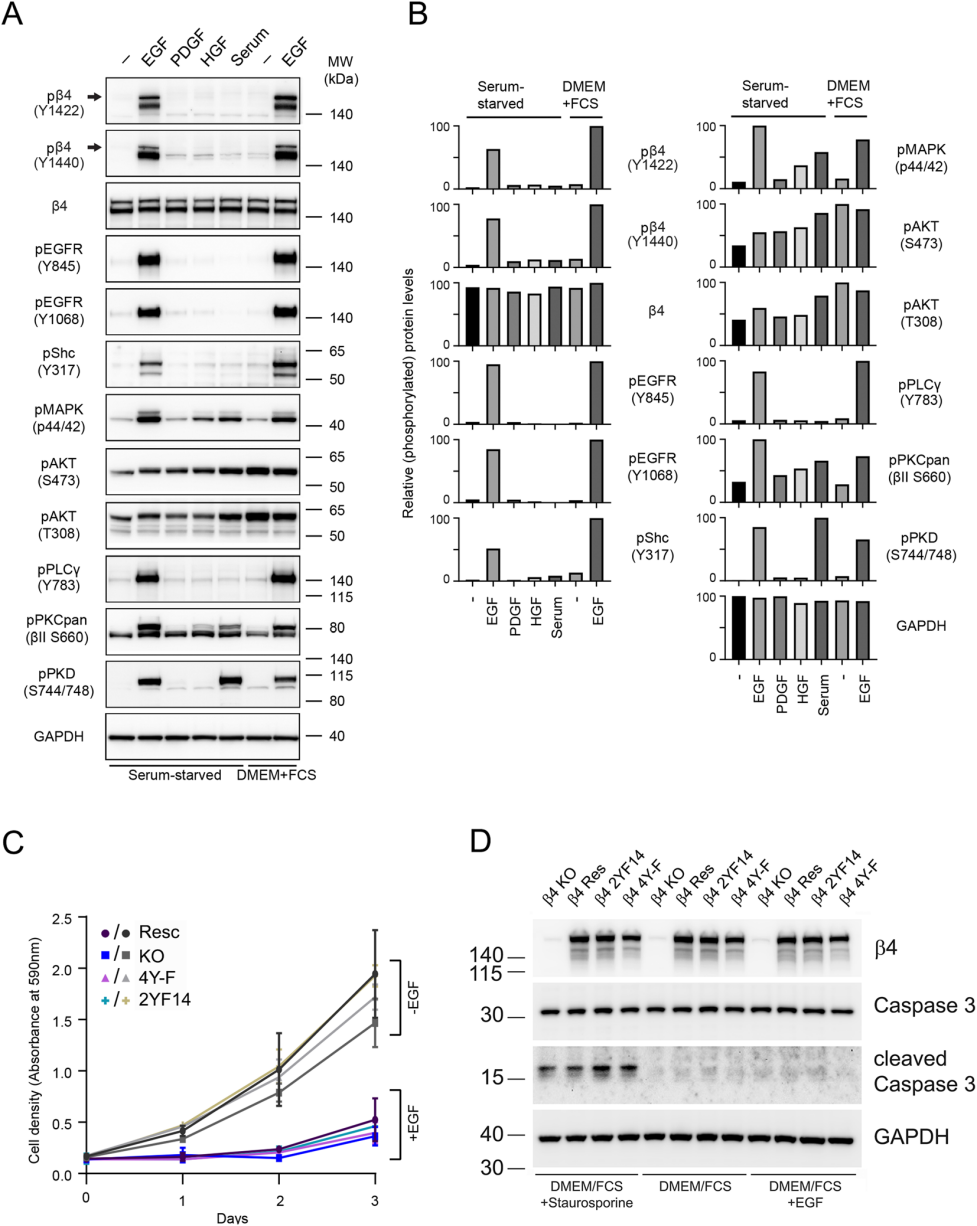
Inhibition of A431 cell proliferation by EGF. (**A**) A431-β4 (KO) cells reconstituted with WT β4 (Resc) grown in DMEM/FCS and serum-starved for 20 h were left untreated or treated with EGF, HGF or PDGF at 50 ng ml^−1^, or 2% FCS for 10 min. Additionally, A431-β4 (Resc) cells grown in DMEM/FCS were left untreated or treated in the presence of serum with 50 ng ml^−1^ EGF. Cell lysates were immunoblotted for pβ4 (Y1422), pβ4 (Y1440), total β4, pEGFR (Y845), pEGFR (Y1068), pShc (Y317), pMAPK (p44/42), pAKT (S473), pAKT (T308), pPLCγ (Y783), pPKC βII (S660), pPKD (S744/748) and GAPDH (loading control). Note, that the lower band of β4 (the precursor or a degradation product of β4) runs at the same height as the phosphorylated EGFR. (**B**) Quantification of Western blot data from (**A**). (**C**) Growth inhibition of A431-β4 (KO) and A431-β4 (Resc) cells and the β4 mutant A431 cell lines A431-β4 (2Y14F) and A431-β4 (4Y-F) by EGF. Cells were seeded in triplicate at 5.000 cells per well in a microtiter plate in DMEM/FCS in the presence or absence of 50 ng ml^−1^ EGF. After adhesion, cell proliferation was assessed by crystal violet staining on four consecutive days. Each point is the mean ± SD from two independent experiments. (**D**) Western blot analyses of total lysates from A431-β4 (Resc) cells and β4 mutant A431 cells, cultured in DMEM/FCS for 24 h in the presence or absence of 50 ng ml^−1^ EGF, for β4, total Caspase-3, cleaved Caspase-3 and GAPDH (loading control). Cells treated for 3 h with 1 μM staurosporine served as positive control of apoptosis induction. Uncropped images of merged chemiluminescent and colorimetric blots obtained with a ChemiDoc imaging system (BioRad) are shown in Suppl. Fig. 6.

Fetal calf serum (FCS)-containing medium is used to grow and maintain A431 cells in the laboratory setting and endogenous bovine EGF within the serum may contribute to the EGFR-β4 signaling axis. Therefore, we next investigated the effects of serum on the activation of the EGFR and phosphorylation of the integrin β4 subunit in A431 cells. Unlike EGF, serum treatment of serum-starved A431-β4 (Resc) cells did not result in a rapid, robust phosphorylation of the EGFR and the integrin β4 subunit at Y1422 and Y1440 (Fig. 6A,B). By contrast, PKC and PKD phosphorylation was strongly induced after serum stimulation which indicates that serum factors other than EGF are sufficient for their induction. Serum treatment also increased the levels of phosphorylated ERK and Akt. However, while EGF stimulated the phosphorylation of ERK more strongly than serum, the opposite effect was observed for Akt phosphorylation. Intriguingly, phosphorylation of PLCγ and Shc was not increased following serum addition, indicating that serum-induced activation of PKC and PKD is not dependent on PLCγ and Shc. Besides serum, we also tested the contribution of HGF and PDGF to intracellular signaling in A431 cells. Treatment with HGF resulted in an increase in the phosphorylation of Akt and ERK1/2, but did not induce the phosphorylation of β4 at Y1422 and Y1440. Consistent with A431 cells lacking the PDGF receptor, these cells also did not respond to the treatment with PDGF.

We also examined the phosphorylation of β4 in A431-β4 (Resc) cells grown in regular growth medium (DMEM supplemented with 10% FCS) both before and after EGF addition. Similar to our findings after serum treatment of serum-starved cells, tyrosine phosphorylation of the EGFR and β4 was not detected in A431-β4 (Resc) cells grown in regular growth medium in the absence of exogenous EGF. However, similar to the serum-starved cells, EGF treatment induces the robust phosphorylation of the EGFR and β4 subunit and increases activation of downstream signaling pathways found downstream of the EGFR. Only, phosphorylation of Akt, a critical effector of the EGFR signaling cascade, was not enhanced, probably because this phosphorylation was already maximal in A431-β4 (Resc) cells grown in regular growth medium. Together these findings indicate that EGF is either absent or the concentration of EGF in the serum is too low to induce EGFR activation and that phosphorylation of β4 at Y1422 and Y1440 does not detectably contribute to the EGFR signaling output in A431-β4 (Resc) cells. Furthermore, these data indicate that serum factors other than EGF are responsible for the increase in ERK, Akt, PKC and PKD phosphorylation observed in A431 cells treated with serum.

Next, we investigated the effect of EGF on the proliferation of the A431-β4 (Resc) cells. In these analyses we also included A431-β4 (KO) cells reconstituted with β4-4YF and -2Y14F to assess possible effects of EGF-induced tyrosine phosphorylation of β4 on cell proliferation. Previous studies have reported a growth inhibitory effect of EGF for A431 cells at doses that are mitogenic in many other cells^47–49^. Consistent with these findings, the addition of EGF to the growth medium strongly decreased the proliferation of A431-β4 (Resc) cells (Fig. 6C). Likewise, proliferation of the mutant cells was inhibited with EGF. Furthermore, no significant changes in apoptosis were observed between control A431-β4 (Resc) cells and A431-β4 (Resc) cells treated with 50 ng/ml EGF for 24 h, and between control A431-β4 (Resc) cells and mutant A431 cells (Fig. 6D). Because the growth inhibitory effects by EGF were similar in the three cell lines, and the cell lines exhibited comparable growth in the absence of EGF, we conclude that β4 phosphorylation by EGFR activation has no role in the proliferation of A431 cells in serum-containing medium.

## Discussion

Both EGFR and β4 are frequently overexpressed in epithelial cancers, and cooperation between EGFR and β4 signaling has been reported to promote tumorigenesis. The ability of integrin α6β4 to amplify and potentiate signals from growth factor receptors is believed to be dependent on specific tyrosine phosphorylation sites in the β4 cytoplasmic domain, which could serve as docking sites for adaptor proteins that link the integrin to PI3K/Akt and Ras/MAPK signaling pathways^11,15,18^. In this paper we investigated the contribution of β4 and four tyrosines in the CS, Y1343, Y1349, Y1422 and Y1440, to EGFR-mediated intracellular signaling. We confirmed the involvement of EGFR overexpression in β4 tyrosine phosphorylation and identified PLCγ1, but not Shc, as a possible binding partner of pY1422 and pY1440. However, in contrast to previous reports^14,16^, we found that SFKs are not involved in β4 tyrosine phosphorylation, and neither β4 nor its tyrosine phosphorylation contribute significantly to EGFR signaling output in cells.

Several studies have shown that β4 can enhance PI3K/Akt signaling downstream of RTKs^43,50–52^. However, in A431 cells, in which Akt phosphorylation is already constitutively high and EGF stimulation only slightly increased Akt phosphorylation, a β4-dependent effect on Akt phosphorylation could not be demonstrated. It is possible that the high level of Akt phosphorylation in A431 cells precluded detection of a subtle effect of β4 on Akt phosphorylation. In line with this reasoning, previous reports have shown that β4 does influence PI3K signaling, but that the effect of β4 on PI3K signaling is diminished by the presence of constitutively active Akt^53,54^. However, in HaCat keratinocytes that do not exhibit high constitutive levels of phosphorylated Akt and in which the EGFR is not overexpressed, an effect of β4 on the EGFR-mediated phosphorylation of Akt could not be demonstrated. The absence of such a β4 effect is not entirely surprising considering the fact that EGF caused only a very modest increase in the tyrosine phosphorylation of β4 in HaCat keratinocytes. Recently, we showed that β4 through its ability to modulate cellular tension can influence Akt phosphorylation^55^. Differences in Akt phosphorylation have also been observed in cells growing in 2D versus 3D^56^. Whether these differences can be related to alterations in cellular tension between the two culture conditions is not known, but they clearly show that mechanisms other than growth factor-induced phosphorylation of β4 can activate Akt.

In addition to PI3K/Akt signaling, β4 tyrosine phosphorylation has been shown to stimulate the Ras/MAPK signaling pathway. In HUVEC cells which transiently co-express α6 together with wild-type or phospho-defective mutants of β4 (Y1526, Y1422 and Y1440), MAPK signaling was shown to be dependent on the phosphorylation of these residues^11,12^. However, using A431 cells, we were unable to demonstrate a role of Y1422 and Y1440 phosphorylation in the EGF-stimulated activation of ERK1/2. The absence of an effect of β4 on MAPK signaling is in agreement with our finding that Shc, which has been shown to be the first step in the activation of the MAPK signaling pathway, does not bind phosphorylated β4 at Y1422 and Y1440^11,16^. Although a role of Y1526 in the activation of MAPK signaling in this study has not been investigated, we consider it unlikely that this residue contributes to EGF-stimulated activation of MAPK since (1) almost all of β4 tyrosine phosphorylation in A431 cells occurs on Y1343, 1349, 1422 and 1440 and (2) the structural environment of Y1526 in FnIII-3 is not compatible with recognition by the SH2 or PTB domain of Shc^46^.

Like many others, we used phosphorylated ERK1/2 as a readout of MAPK pathway activation. Although we found no evidence that tyrosine-phosphorylated β4 plays a role in the activation of ERK1/2, alternative readouts of MAPK signaling could provide additional information. For example, previous work in vivo has shown that the phosphorylation of ERK1/2 does not change but instead the translocation of ERK1/2 to the nucleus differed between cells expressing wild-type β4 and a β4 mutant lacking its signaling domain (truncated after 1355)^57^. Since nuclear translocation of ERK1/2 is required for regulation of the cell cycle and proliferation, but not for activation of other downstream kinases such as RSKs, MSKs and MNKs or phosphorylation of other cytoplasmic targets (e.g. β4 itself^58^), our data does not exclude a contribution of β4 downstream of ERK1/2 activation to gene expression via the MAPK pathway.

In addition to the MAPK/ERK1/2 and PI3K/Akt signaling pathways, EGF stimulates the PLCγ1/PKC pathway. We identified PLCγ1 as a possible binding partner of tyrosine phosphorylated β4 by peptide pulldowns but were unable to confirm this interaction within a cellular environment by co-immunoprecipitation. Moreover, the absence of β4 or its tyrosine phosphorylation in A431 cells also did not affect the activation of PLCγ1 or its downstream effectors PKC and PKD. These results indicate that although the phosphorylated residues Y1422 and Y1440 in β4 can provide docking sites for PLCγ1, binding of PLCγ1 to β4 is not needed for efficient PLCγ1/PKC signaling downstream of the EGFR. PKC and PKD are activated not only by PLCγ downstream of RTK activation, but also by GPCR-mediated PLCβ activation. Because we were unable to detect EGFR and PLCγ activation by serum stimulation of A431 cells, it is possible the activation of PKC/PKD activation in these cells occurs downstream of GPCR activation. Similarly, GPCR activation might be responsible for the increase in Akt phosphorylation upon serum stimulation, while the increase in ERK1/2 activation is mediated by PKC downstream of PLCβ activation.

Our finding that β4 tyrosine phosphorylation does not significantly contribute to growth factor mediated signaling is consistent with findings reported by Merdek et al.^45^. These authors expressed a chimeric receptor containing the β4 cytoplasmic domain in MDA-MB-435 breast cancer cells and showed that the β4 chimera was tyrosine phosphorylated upon stimulation with hepatocyte growth factor (HGF), but did not enhance HGF-induced Akt and ERK1/2 phosphorylation^45^. Furthermore, several studies showed that α6β4 ligation with antibodies or ligand results in the activation of SFKs and subsequent Akt and ERK1/2 signaling^15,16,18^ and that β4 signaling is only relevant when growth factors and hormones are limited by low availability^54,59^. However, because β4 is minimally tyrosine phosphorylated in cells that do not overexpress the EGFR and stimulation of these cells with suboptimal concentration of EGF will only further decrease its phosphorylation level, we do not consider it likely that β4 would make a significant contribution to EGFR signaling in such conditions. Moreover, in A431 cells that overexpress the EGFR and exhibit high levels of tyrosine phosphorylated β4, EGF stimulation causes growth arrest of the cells. This phenomenon has been previously observed and suggests that the low level of ligand-independent activation of the overexpressed EGFR in A431 cells is sufficient to support their growth^47–49,60^. Thus, the EGF-induced tyrosine phosphorylation of β4 in A431 cells might be the result of the abnormal high levels of RTK activation in these cells and have no functional significance.

Previously the involvement of SFKs in tyrosine phosphorylation of β4 was shown by others^21,36^. Our data shows that, in contrast to results from the Giancotti lab^21^, tyrosine phosphorylation of β4 is not dependent on any one of the highly expressed SFKs Src, Fyn, Yes or Lyn. Even when expression of Fyn, Yes and Lyn were simultanously silenced, there was hardly any change in the level of β4 tyrosine phosphorylation. However, in agreement with the Giancotti lab, we found that the SFK inhibitor PP2 inhibitor greatly reduced EGF-induced β4 tyrosine phosphorylation. Although PP2 is a potent and selective inhibitor of SFKs, it can inhibit other kinases with sufficient potency, which could contribute to tyrosine phosphorylation of β4, e.g. CSK and PTK6^61,62^. Furthermore, it is known that PP2 weakly inhibits EGFR, and thus it is possible that the EGFR itself directly phosphorylates β4. In this regard it is interesting to note that the tyrosine residues Y1343, Y1422 and Y1440 lie within an EGFR kinase consensus sequence (X[E/D]pYX) and that β4 and EGFR are often found in close proximity of each other. Alternatively, inhibition of EGFR by PP2 could reduce the activation of multiple kinases downstream of the EGFR important for β4 tyrosine phosphorylation.

In summary, we investigated whether tyrosine phosphorylation of β4 is contributing to EGFR induced intracellular tumorigenic signaling in epithelial cells. Combining our data with data from others, we conclude that, although β4 can contribute to PI3K or MAPK signaling under certain circumstances, EGF-induced tyrosine phosphorylation of β4 does not substantially contribute to pro-tumorigenic signaling downstream of EGFR and therefore the biological relevance of EGF-induced tyrosine phosphorylation of β4 remains unknown.

## Materials and methods

### Reagents

The primary and conjugated antibodies used in this study are listed in Table S1. Secondary antibodies used for Western blot were goat anti-mouse IgG HRP (BioRad; 1:3000) and polyclonal goat anti-Rabbit IgG HRP (Dako; 1:5000). Secondary antibodies used for immunofluorescence were donkey anti-rabbit IgG Alexa 594 (Invitrogen A21207; 1:400), goat anti-mouse IgG Alexa 568 (Invitrogen A11004; 1:200) and goat anti-guinea pig Alexa 488 (Invitrogen A11073; 1:200). The PP2 and PP3 compounds were purchased from Merck Chemical Ltd. EGF and PDGF-BB were obtained from Sigma-Aldrich. HGF was obtained from R&D Systems and staurosporine was from Tocris Bioscience. The polyclonal rabbit antibodies against the phosphorylated Y1440 and Y1422 sites on β4 are homemade (method described in supplemental materials).

### Cell culture and preparation of cell lines

Cells used are PA-JEB, PA-JEB/β4, HaCaT, A431, HT29, COS7, DiFi, MDA-MB-231, MCF10A and MDA-MB-468. Detailed information about culture media, growth conditions and origin can be found in the supplemental materials. Cells were modified using CRISPR-Cas9 mediated KO, siRNA-mediated KD, stable (re)expression of proteins by retroviral transduction and transient overexpression of proteins. The detailed methods and cDNA constructs used are described in the supplemental materials.

### Flow cytometry/FACS

Flow Cytometry/FACS was performed essentially as described previously^63^. Cells were collected after trypsinization, and incubated for 50–60 min with conjugated β4-PE antibody on ice in PBS containing 2% FCS. After incubation, cells were washed twice with 2% FCS in PBS. Finally, cells were passed through a nylon mesh filter and 50,000 positive cells were analyzed per sample using a FACSCalibur cell analyzer (BD Biosciences). β4 KO cells were used as a negative control. The graphs were made in FlowJo and adapted in Adobe Illustrator.

### Immunofluorescence imaging

Immunofluorescence imaging was performed essentially as described previously^63^. Cells were seeded on glass coverslips and grown for 40 h in the presence of DMEM + FCS. Cells were fixed wih 2% PFA for 10 min, permeabilized with 0.2% Triton X-100 and blocked with 2% BSA (SERVA) in PBS. Cells were incubated with primary and secondary antibodies for 50–60 min. In between and after, cells were washed three times with PBS. Nuclei were stained with DAPI (Sigma-Aldrich) and MOWIOL was used to mount the coverslips for confocal imaging. Imaging was performed using a Zeis LSM 980 Airyscan 2 confocal microscopy with a 63× (NA 1.4) oil objective (Plan-Apochromat SF25, Zeiss). Raw images were taken on the Airyscan in Multiplex mode (SR-4Y) and processed using the Zen blue software’s Airyscan.

### Immunoprecipitations and western blot

For analysis of proteins in whole cell lysates, subconfluent cells were washed in cold PBS, lysed in 1% Nonidet P-40, 100 mM NaCl, 4 mM EDTA, 20 mM Tris–HCl (pH 7.5), supplemented with 1.5 mM Na_3_VO_4_, 15 mM NaF as phosphatase inhibitors and a protease inhibitor cocktail (1:500; Sigma), whole cell lysates were cleared by centrifugation at 12,000 × *g* for 60 min at 4 °C and supplemented with SDS sample buffer (50 mM Tris–HCl pH 6.8, 2% SDS, 10% glycerol, 12.5 mM EDTA, 0.02% Bromophenol Blue) with β-mercaptoethanol and heated at 95 °C for 5 min^63^.

Immunoprecipitations were performed with the cleared cell lysates after centrifugation. The lysates were incubated at 4 °C for 1.5–2 h with 1 µg antibody. Subsequently, the lysates were incubated 4–20 h at 4 °C with prewashed Protein G Sepharose 4 Fast Flow beads (GE Healthcare), beads were washed two times with lysis buffer and two times with PBS and bound proteins were dissolved in SDS sample buffer with β-mercaptoethanol and heated at 95 °C for 5 min. Proteins were separated on 4–12% Bolt gradient gels (Invitrogen) and transferred to Immobilon-P transfer membranes (Millipore). The membrane was blocked for at least 2 h in 2% BSA in TBST (10 mM Tris (pH 7.5), 150 mM NaCl, 0.05% Tween 20) before incubation with primary antibody overnight at 4 °C and with secondary antibody for 1 h at room temperature. After each incubation step, the membranes were washed twice with TBST and twice with TBS (TBST without Tween 20) as described previously^64^. Antibodies were detected using Clarity Western ECL Substrate (Bio-Rad). Quantification of western blot data was performed with ImageJ.

### RT-PCR

Reverse transcriptase–quantitative polymerase chain reaction (RT-qPCR) analysis was performed as described previously^64^. Reactions were performed in triplo for determination of the levels of Yes, Lyn, Frk, Lck, Fgr, Hck, Blk and CyclofilinA (CF; control) in A431 cells. Cells were grown in complete medium and lysed in RNA-Bee (Tel-test Inc.) or Trizol reagent (Ambion #15596018), following manufacturer’s instructions. Total RNA was separated from DNA and proteins by the addition of chloroform (Sigma-Aldrich) and subsequent centrifugation at 12,000 × *g* for 15 min at 4 °C. The RNA was precipitated with isopropanol and washed with 70% ethanol. Integrity of the isolated RNA was assessed by agarose gel electrophoresis.

First strand cDNA synthesis was performed with 3 μg of total RNA using the first strand cDNA synthesis kit K1612 (Thermo Fisher Scientific) following manufacturer’s directions. The PCR reactions were run using SYBR Advantage qPCR premix (Clontech) on a 7500 Fast Real-Time PCR system (Applied Biosystems) and the primers (IDT) which were used, are shown in Table S2. Relative mRNA quantities were obtained using the 2^−ΔCt^ method. The ΔCt is the obtained Ct value of the SFK minus the obtained Ct value of CF (endogenous control).

### Peptide pulldowns and mass spectrometry analyses

Phosphorylated and unphosphorylated peptides based on sequences within the integrin β4 (Table S3) were synthesized by Fmoc chemistry in the laboratory of Huib Ovaa at the NKI and purified by HPLC. The peptides were synthesized with a cysteine followed by a caproic acid at the amino terminus. The cysteine was used for coupling to SulfoLink (Pierce, Rockford, IL), while the caproic acid was added to provide spacing between the peptide and the support material. The sequence of each peptide was verified by mass spectrometry.

For peptide pull downs, PA-JEB/β4 keratinocytes were grown to confluency on 15 cm tissue culture dishes, lysed in 3 ml 50 mM HEPES pH 7.5, 150 mM NaCl, 10% glycerol, 1% Triton X-100, 1.5 mM MgCl_2_, 1 mM EGTA, 100 mM NaF, 10 mM sodium pyrophosphate, 500 μM sodium vanadate, 10 μg ml^−1^ aprotinin and 10 μg ml^−1^ leupeptin (PLC-lysis buffer) per 15 cm tissue culture dish. Lysates were precleared by centrifugation at 10,000 rpm in a microfuge at 4 °C for 30 min. and incubated for 1 h with 100 μl of a 10% slurry of each of the different β4 peptides. Agarose beads were collected by centrifugation and washed four times with PLC buffer. Sample were boiled, resolved by SDS-PAGE and stained with Coomassie blue. Protein bands of interest were cut out, subjected to in-gel trypsin digest and analyzed by mass spectrometry.

### Proliferation assay

Cells were seeded in 96 well plates at a density of 5000 cells per well. Cells were collected for 4 consecutive days. The cells were washed with PBS, fixed with 2% paraformaldehyde for 10 min, washed with PBS and stained with 2% crystal violet for 10 min. After washing with demiwater, plates were dried overnight and cells were lysed in 2% SDS. Absorbance was measured at 590 nm on an Epoch microplate spectrophotometer (BioTek).

## Supplementary information

Supplementary Informations.
