# Supplementary material for: EGFR-dependent tyrosine phosphorylation of integrin β4 is not required for downstream signaling events in cancer cell lines
Source: Sci Rep. 2021 Apr 21;11:8675. doi: 10.1038/s41598-021-88134-6 (PMC8060419; doi:10.1038/s41598-021-88134-6)
Supplement: Supplementary file 1 — Supplementary Informations. [file 41598_2021_88134_MOESM1_ESM.pdf]

Supplementary Information

EGFR-dependent tyrosine phosphorylation of integrin β4 is not required for downstream signaling events in cancer cell lines

Lisa te Molder, Maaike Kreft, Niels Heemskerk, Joyce Schuring, Jose de Pereda, Kevin Wilhelmsen, Arnoud Sonnenberg.

Supplemental information – Materials and Methods

Table S1 Primary antibodies used in various techniques.

Target	Antibody	Application	Source
α-tubulin	Mouse mAb	WB (1:10000)	Sigma #t5168
Caspase-3	Rabbit pAb	WB (1:1000)	Cell Sign. #9662
Cleaved Caspase-3 (D175)	Rabbit pAb	WB (1:1000)	Cell Sign. #9661
EGFR	Mouse mAb 528	IF (1:50), IP (1 μg ml^{-1})	Calbiochem
EGFR	Rabbit pAb 1005	WB (1:250)	Santa Cruz #sc-03
Fyn	Rabbit pAb	WB (1:1000)	Cell Sign. #4023P
GAPDH	Rabbit mAb D16H11	WB (1:1000)	Cell Sign. #5174S
Itg. β1	Mouse mAb TS2/16	IP (1 μg ml^{-1})	ATCC
Itg. β4	Mouse mAb 450-11A	IF (1:500), IP (1 μg ml^{-1})	Kind gift from Steve Kennel
Itg. β4	Rabbit pAb	IF (1:2000)<br>WB (1:2000)	Homemade
Itg. β4	PE-Rat anti-human-CD104	FACS (1:400)	BD Pharm. #555720
Itg. pβ4 (Y1440)	Rabbit pAb	WB	Homemade
Itg. pβ4 (Y1422)	Rabbit pAb	WB	Homemade
Lyn	Mouse mAb H-6	WB (1: 500)	Santa Cruz #sc-7274
MAPK	Mouse mAb	WB (1:1000)	BD Trans. #610104
pAkt (S473)	Rabbit pAb	WB (1:500)	Cell Sign. #9271S
pAkt (T308)	Rabbit pAb	WB (1:1000)	Cell Sign. #9275
pEGFR (Y845)	Rabbit pAb	WB (1:1000)	Cell Sign. #2231
pEGFR (Y1068)	Mouse mAb	WB (1:1000)	Cell Sign. #2236S
Plcγ1	Rabbit pAb 530	WB (1:200)	Santa Cruz #sc-426
pMAPK (p44/p42)<br>(T202/Y204)	Rabbit pAb	WB (1:1000)	Cell Sign. #9101
pPKCα,β II (T638/641)	Rabbit pAb	WB (1:1000)	Cell Sign. #9375
pPKC (pan) (βII S660)	Rabbit pAb	WB (1:1000)	Cell Sign. #9371S
pPKD (S744/748)	Rabbit pAb	WB (1:1000)	Cell Sign. #2054S
pPlcγ1 (Y783)	Rabbit mAb D6M9S	WB (1:1000)	Cell Sign. #14008S
pShc (Y317)	Rabbit pAb	WB (1:1000)	Cell Sign. #2431
pTyrosine (pY)	Mouse mAb 4G10	WB (5 μg ml^{-1})	Millipore Sigma
Shc	Mouse mAb	WB (1:1000)	BD Trans. #S68020
Src	Rabbit mAb 32G6	WB (1:1000)	Cell Sign. #2123
Yes	Mouse mAb	WB (1:1000	BD Trans. #610375

Antibodies against pY1440 and pY1422

The polyclonal rabbit antibodies against the phosphorylated Y1440 and Y1422 sites on β4 were raised against synthetic peptides with the sequence SSTLTRD**pY**NSLTRSE and STTLPRD**pY**STLTSVS respectively. The two synthetic peptides were conjugated to maleimide-activated mcKLH and injected into rabbits. The rabbits received a booster immunization every 4 weeks and antisera were collected 1 week after the third booster. To prevent recognition of other epitopes by the antibody during immunoblotting, the antibodies were used in combination with 10 μM of both unphosphorylated synthetic peptides for β4 and 20 μM phosphorylated EGFR Y1068 peptides.

Cell culture

PA-JEB immortalized keratinocytes were isolated from a patient with Pyloric Atresia associated with Junctional Epidermolysis Bullosa [1,2]. PA-JEB/β4 keratinocytes stably expressing wild-type β4 were generated by retroviral transduction [3] and maintained in serum-free keratinocyte medium (KGM; Invitrogen), supplemented with 50 μg ml^{-1} bovine pituitary gland extract, 5 ng ml^{-1} EGF, and antibiotics (100 units ml^{-1} streptomycin and 100 units ml^{-1} penicillin; Sigma-Aldrich). HaCaT keratinocytes, A431 epidermal squamous carcinoma cells, HT29 colorectal adenocarcinoma cells and COS7 monkey kidney cells were obtained from the American Type Culture Collection (ATCC) and

cultured in Dulbecco's modified Eagle's medium (DMEM; Gibco) containing 10% heat-inactivated fetal calf serum (FCS; Serana Europe GmbH, Pessin, Germany), and antibiotics. DiFi rectal carcinoma and MDA-MB-231 breast adenocarcinoma were kindly provided by the laboratories of Rene Bernards and Jos Jonkers at the Netherlands Cancer Inst. (Amsterdam, the Netherlands) cell lines and were cultured in RPMI medium 1640 (Gibco #52400-025) containing 10% heat-inactivated fetal calf serum (FCS; Serana), and antibiotics. MCF10A mammary epithelial cells (obtained from ATCC) were cultured in HAM's medium; DMEM/F12 (Gibco #31331-028) supplemented with 5% horse serum (Sigma-Aldrich), EGF (20 ng ml^{-1}; Sigma-Aldrich), insulin (10 µg ml^{-1}; Sigma-Aldrich), hydrocortisone (0.5 mg ml^{-1}; Sigma-Aldrich), cholera toxin (100 ng ml^{-1}; Sigma-Aldrich) and antibiotics and MDA-MB-468 mammary adenocarcinoma cells (a kind gift from Jos Jonkers lab) in Leibovitz's L-15 medium (Gibco #11415-049) supplemented with 10% heat-inactivated fetal calf serum and antibiotics. All cells were cultured at 37°C in a humidified, 5% CO_2 atmosphere. When cells were serum starved for experimental reasons, the cells were washed two times with PBS and subsequently grown for 12-20h in medium without FCS or growth factors.

Preparation of cell lines

CRISPR-Cas9 mediated KO: The A431 and HaCaT β4 KO cells were prepared using CRISPR-Cas9 technology. Target sgRNAs against integrin β4 (5'-GACTCGCTCCCTCCGCGCCT-3') were cloned in the pX330-U6-Chimeric_BB-CBh-hSpCas9 (Addgene plasmid #42230, deposited by Feng Zhang). The cells were transiently transfected with this plasmid using lipofectamine® 2000 (Invitrogen) in OptiMEM (Gibco), and bulk sorted, for the β4 negative population, using a Moflo Asterios (Beckman Coulter) or FACSAria Fusion (BD Biosciences) cell sorter.

siRNA KD of SFKs in A431 cells: The Yes (SI00302218) and Lyn (SI006605570) siRNAs were purchased from QIAGEN. The human Src (L-003175-00-0005) and Fyn (L-003140-00-0005) SMARTpools and non-targeting control pool (D-001810-10-05) were from Dharmacon. A431 cells were transiently transfected with the siRNAs using lipofectamine® 2000 (Invitrogen). Lipofectamine and siRNA solutions in OptiMEM (Gibco) were mixed and incubated for 20-30 min at room temperature. Cells were incubated with the transfection solution for approximately 48 h. The cells were serum starved for 20 h, by two PBS washes and the replacement of the full medium for medium without supplements and serum, after which the cells were treated with EGF.

Stable (re)expression of proteins by retroviral transduction: For (re)expression of β4 wt and β4 mutants in β4 deficient A431 cells, we used stable cellular retroviral mediated transduction. Virus was produced by calcium phosphate transfection of Phoenix-ampho cells following the retroviral producer line protocol, and added to A431-β4 (KO) cells. Cells which expressed the protein of interest were enriched by zeocin selection and bulk sorted by FACS. The constructs used were β4 WT, β4 4Y-F, β4 2Y14F and β4 2Y13F in LZRS-IRES-Zeo.

Transient overexpression of proteins in COS7 cells: COS7 cells were transfected using the DEAE-dextran method. The constructs used were β4 WT, β4 2Y14F and β4 4Y-F in pcDNA3.

cDNA constructs: The full-length β4 cDNA in pUC18 has been described previously [3]. Point mutants of β4 Y1343, Y1349, Y1422 or Y1440 were generated by site-directed mutagenesis with the PCR-based overlap extension method using Pwo DNA polymerase (Roche Molecular Biochemicals, Indianapolis, IN), and fragments containing the different mutations were exchanged with corresponding fragments in the β4 pUC18 vector. Retroviral vectors containing mutant β4 cDNAs were generated by subcloning the mutant β4 cDNAs into the *Eco*RI restriction site of the pcDNA3 vector or the LZRS-MS-IRES-ZEO vector as described before [4].

References

1. Niessen, C.M., van der Raaij-Helmer, M.H., Hulsman, E.H., van der Neut, R., Jonkman, M.F., & Sonnenberg, A. Deficiency of the integrin beta4 subunit in junctional epidermolysis bullosa with

pyloric atresia: consequences for hemidesmosome formation and adhesion properties. *J. Cell Sci*. **109**, 169-1706 (1996).

2. Schaapveld, R.Q., Borradori, L., Geerts, D., van Leusden, M.R., Kuikman, I., Nieveers, M.G., Steenbergen, R.D., Snijders, P.J. & Sonnenberg, A. Hemidesmosome formation is initiated by the beta4 integrin subunit, requires complex formation of beta4 and HD1/plectin, and involves a direct interaction between beta4 and the bullous pemphigoid antigen 180. *J. Cell Biol*.**142**, 271-284 (1998).
3. Niessen, C.M., Hulsman, E.H., Rots, E.S., Sánchez-Aparicio, P.& Sonnenberg, A. Integrin alpha 6 beta 4 forms a complex with the cytoskeletal protein HD1 and induces its redistribution in transfected COS-7 cells. *Mol. Biol. Cell* **8**, 555-566 (1997).
4. Sterk, L.M., Geuijen, C.A., Oomen, L.C., Calafat, J., Janssen, H. & Sonnenberg, A. The tetraspan molecule CD151, a novel constituent of hemidesmosomes associates with the integrin alpha6beta4 and may regulate the spatial organization of hemidesmosomes. *J. Cell Biol*. **149**, 969-982 (2000).

Table S2 RT-PCR primers

Target	Forward primer sequence	Reverse primer sequence
Yes	5’-CCTCATTTTCAGTGGTGCCA-3’	5’-TGCTTCCCACCAATCTCCTT-3’
Lyn	5’-ACAGCTTGAGTGACGATGGA-3’	5’-ATTCTCCATGCTCCTCCAGG-3’
Lck	5’-GGGATCGTTTTCACTGTCGG-3’	5’-GCCCATCTGAAGCATTGGTG-3’
Fgr	5’-CATTCCCAGCAACTACGTGG-3’	5’-GACAGGGAGTAGGCACCTTT-3’
Hck	5’-TAGCCACAACAGCAACACAC-3’	5’-CACTCCCCGGATTCCTCTAG-3’
Blk	5’-AATGATCGGGACCTGCAGAT-3’	5’-GGCCTTGTTGATTGGAGCAA-3’
CF (control)	5’-CATCTGCACTGCCAAGACTGA-3’	5’-TTGCCAAACACCACATGCTT-3’

Table S3 β4 peptides used for peptide pulldowns

Tyrosine site	Unphosphorylated	Phosphorylated
1422	Cys-Caproic acid-SSTLTRDYNSLTRSE	Cys-Caproic acid-SSTLTRDpYNSLTRSE
1440	Cys-Caproic acid-STTLPRDYSTLTSVS	Cys-Caproic acid-STTLPRDpYSTLTSVS
1494	Cys-Caproic acid-CERPLQGYSVEYQLL	Cys-Caproic acid-CERPLQGpYSVEYQLL
1526	Cys-Caproic acid-DLLPNHSYVFRVRAQ	Cys-Caproic acid-DLLPNHSpYVFRVRAQ

Suppl. Fig. 1: Uncropped images of merged chemiluminescent and colorimetric blots in Fig. 1A

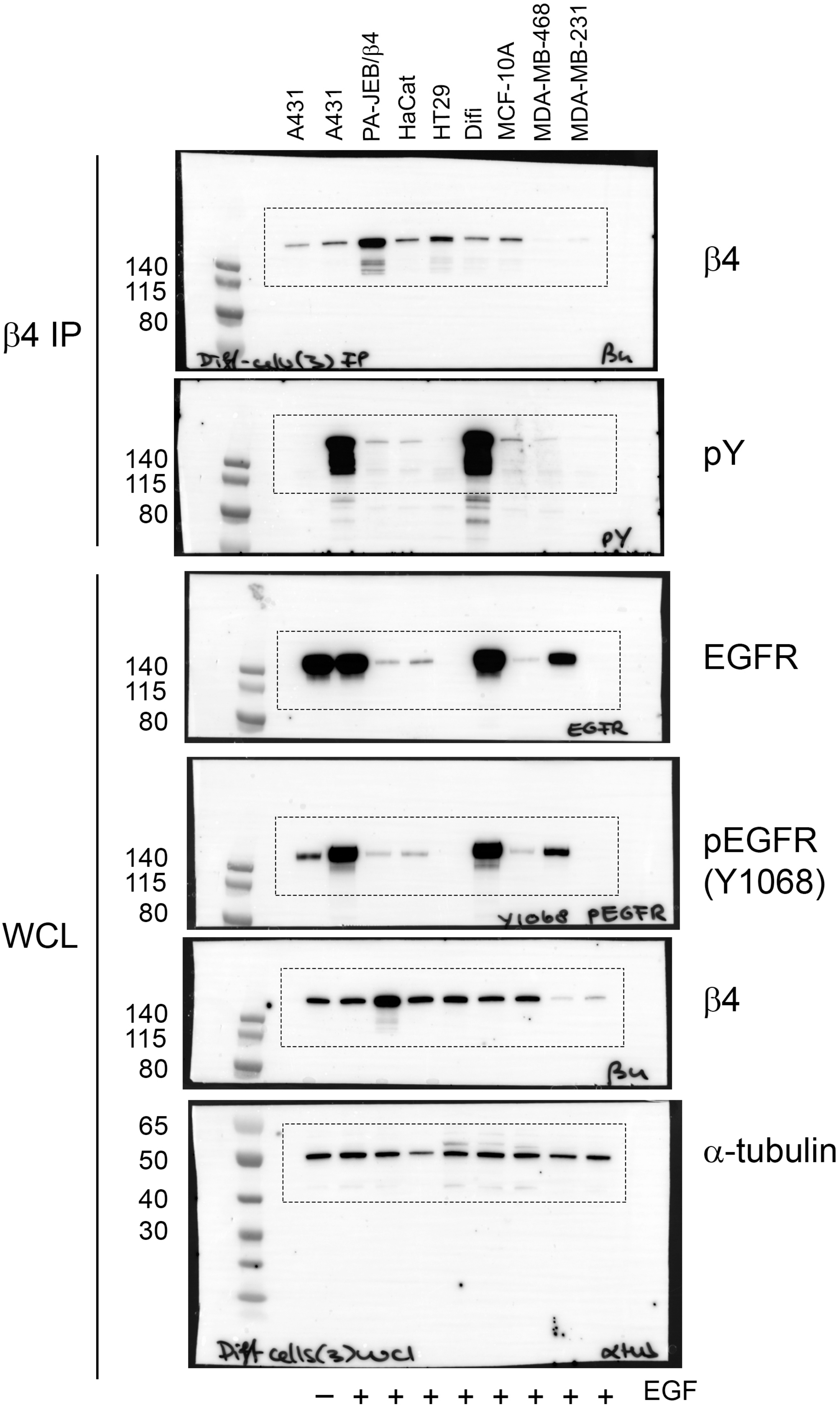

Suppl. Fig. 2: Uncropped images of Western blots in Fig. 2B and merged chemiluminescent and colorimetric blots in Fig. 2D

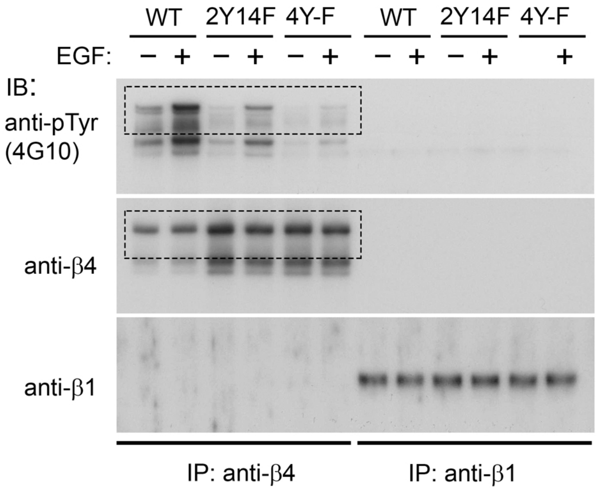

Fig. 2B

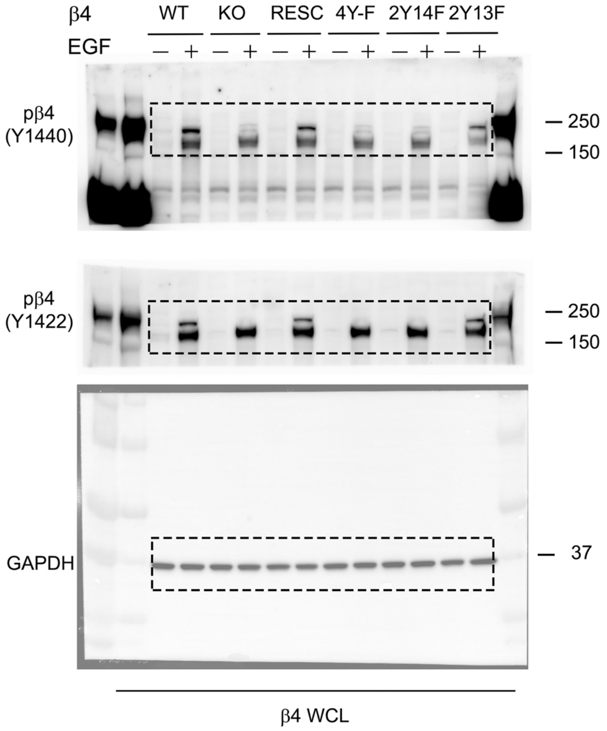

Fig. 2D

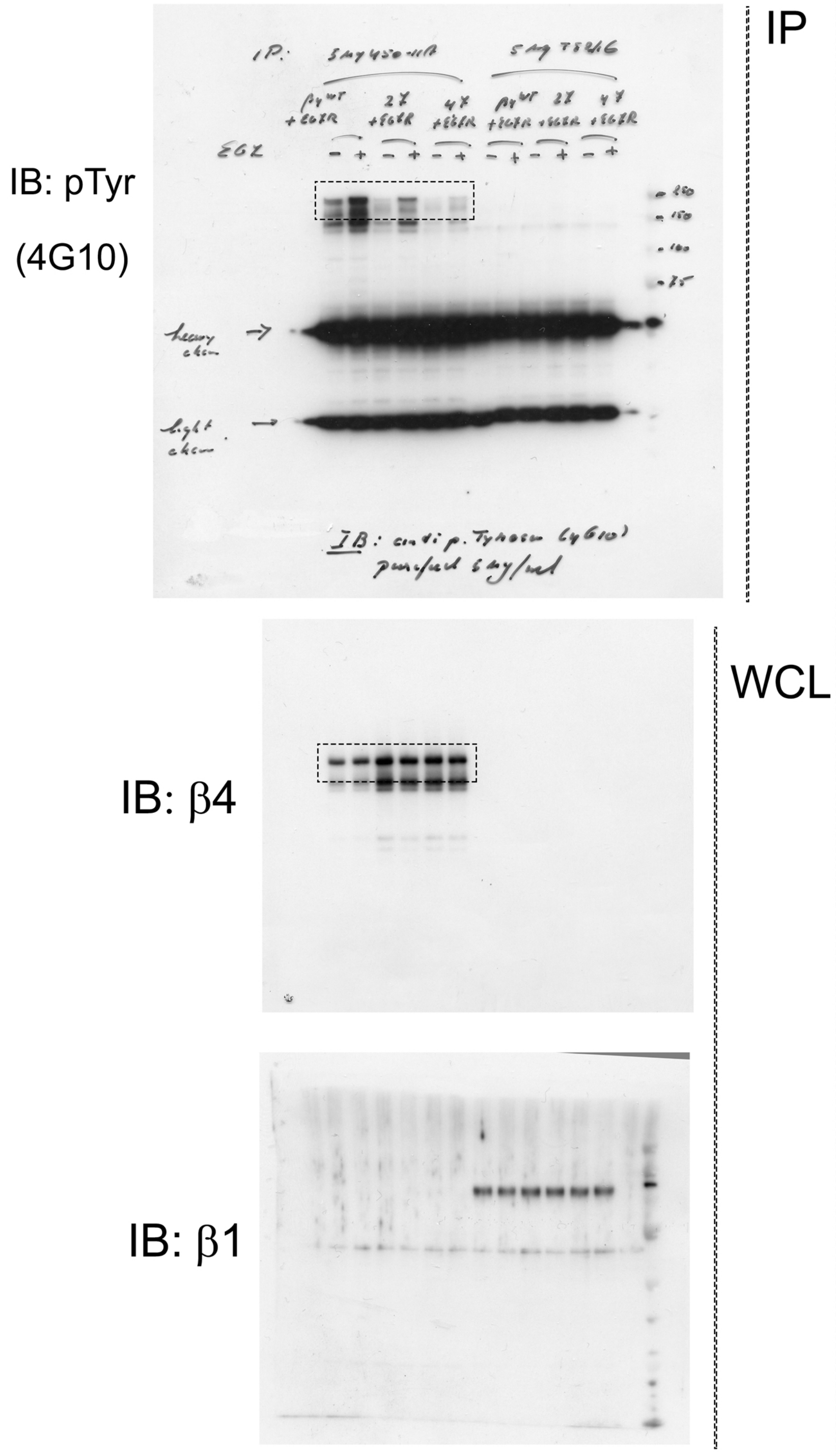

Complete blots of Fig. 2B

Suppl. Fig. 2: Uncropped images of Western blots in Fig. 2F

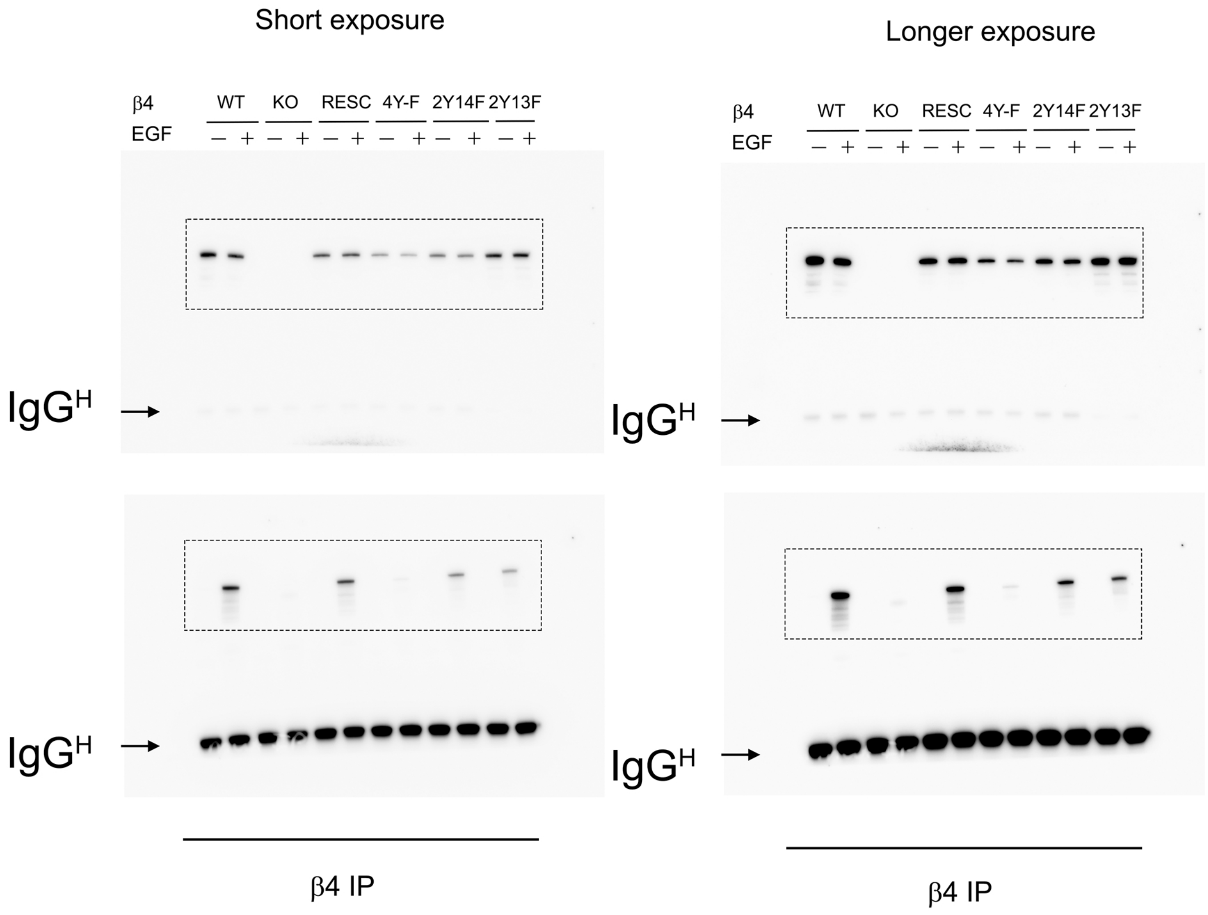

Fig. 2F

Suppl. Fig. 3: Uncropped images of Western blots in Fig. 3A repetition of the experiment in Fig. 3A

Fig. 3A

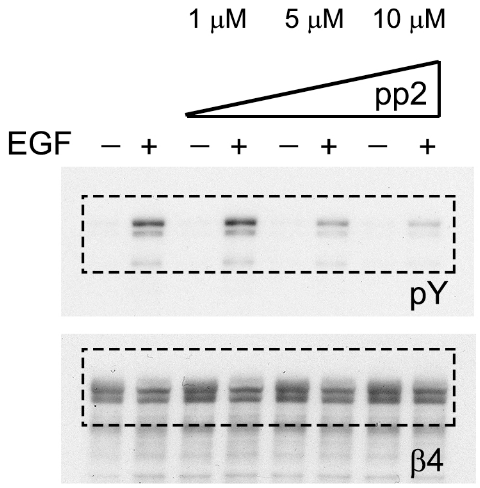

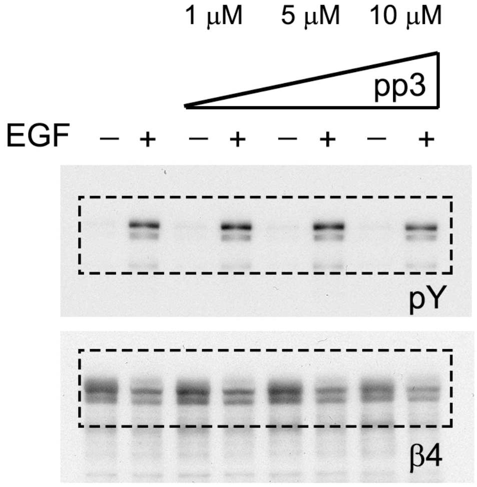

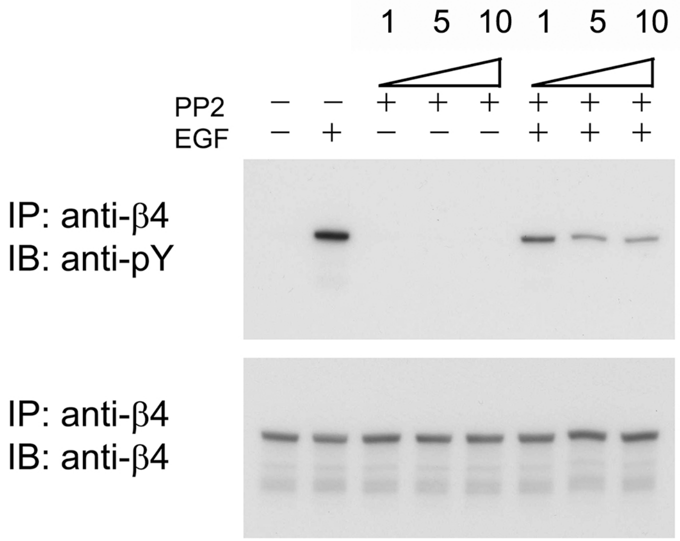

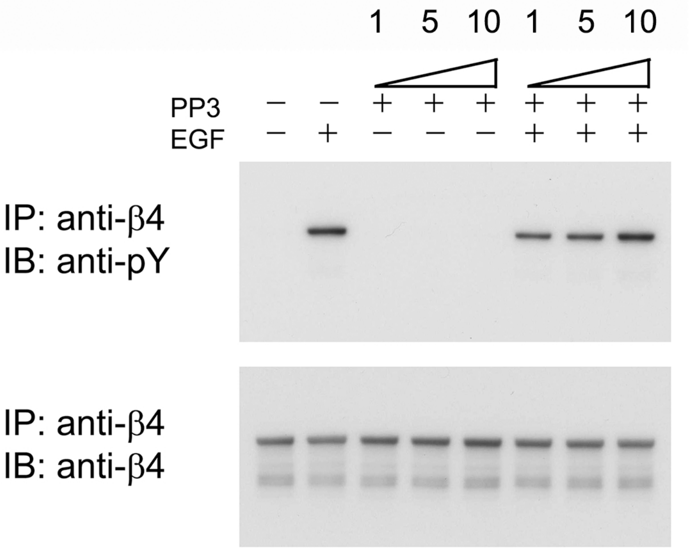

Repetition of the experiment presented in Fig. 3A with A431 cells treated for 5 min with EGF in the presence or absence of PP2 or PP3 (0, 1, 5 and 10 μM). Integrin β4 was immunoprecipitated with mAb 450-11A and blots were probed with anti-pTyr (4G10) and anti β4 (Rabbit pAb). Note that in this experiment wild-type A431 cells were used and not A431 cells overexpressing β4-GFP as in the experiment presented in Fig. 3A

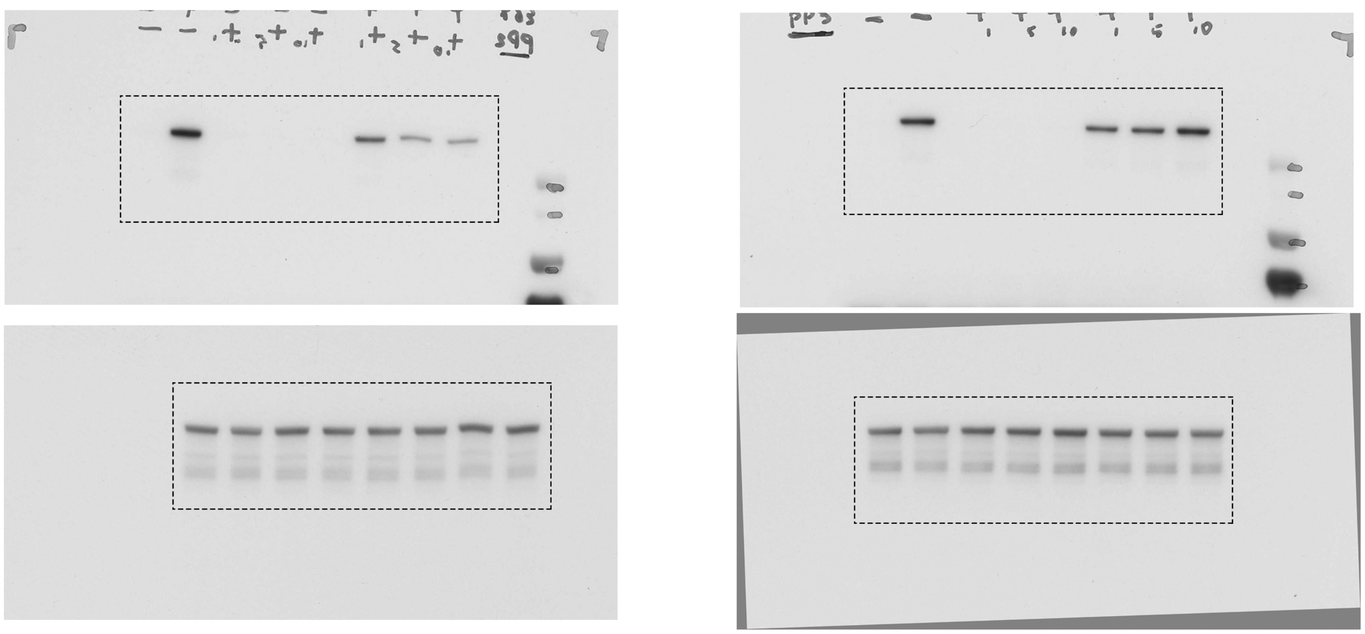

Suppl. Fig. 3: Uncropped images of merged chemiluminescent and colorimteric blots in Fig. 3B and D.

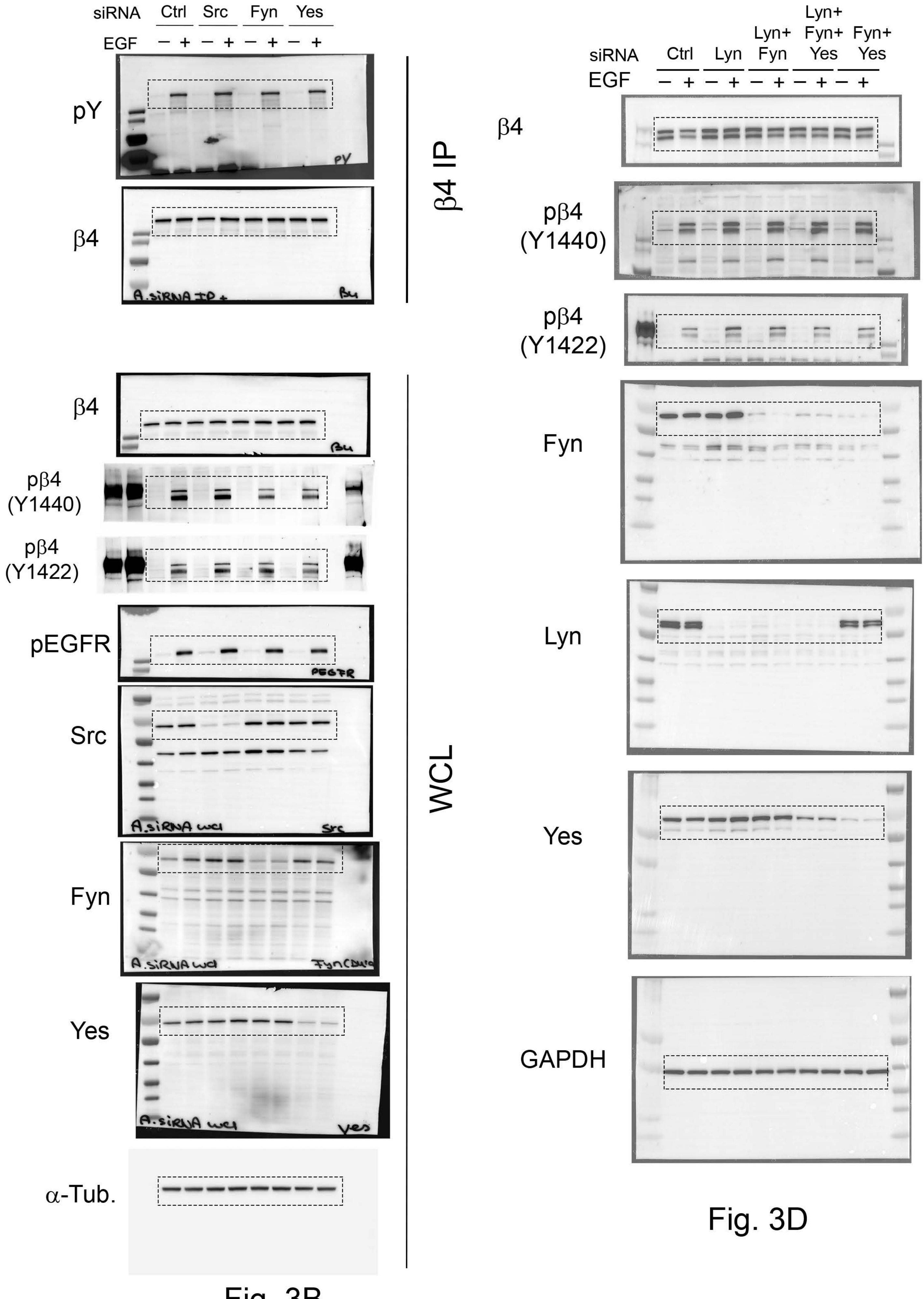

Fig. 3B

Fig. 3D

Suppl. Fig. 4: Uncropped images of Western blots in Fig. 4B and C

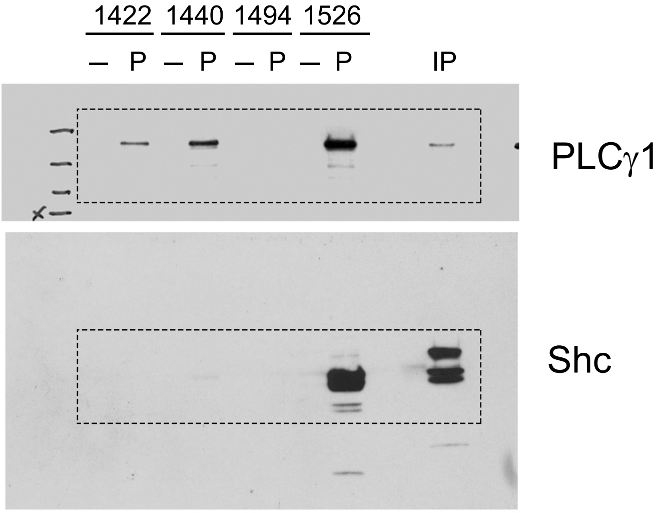

Fig. 4B

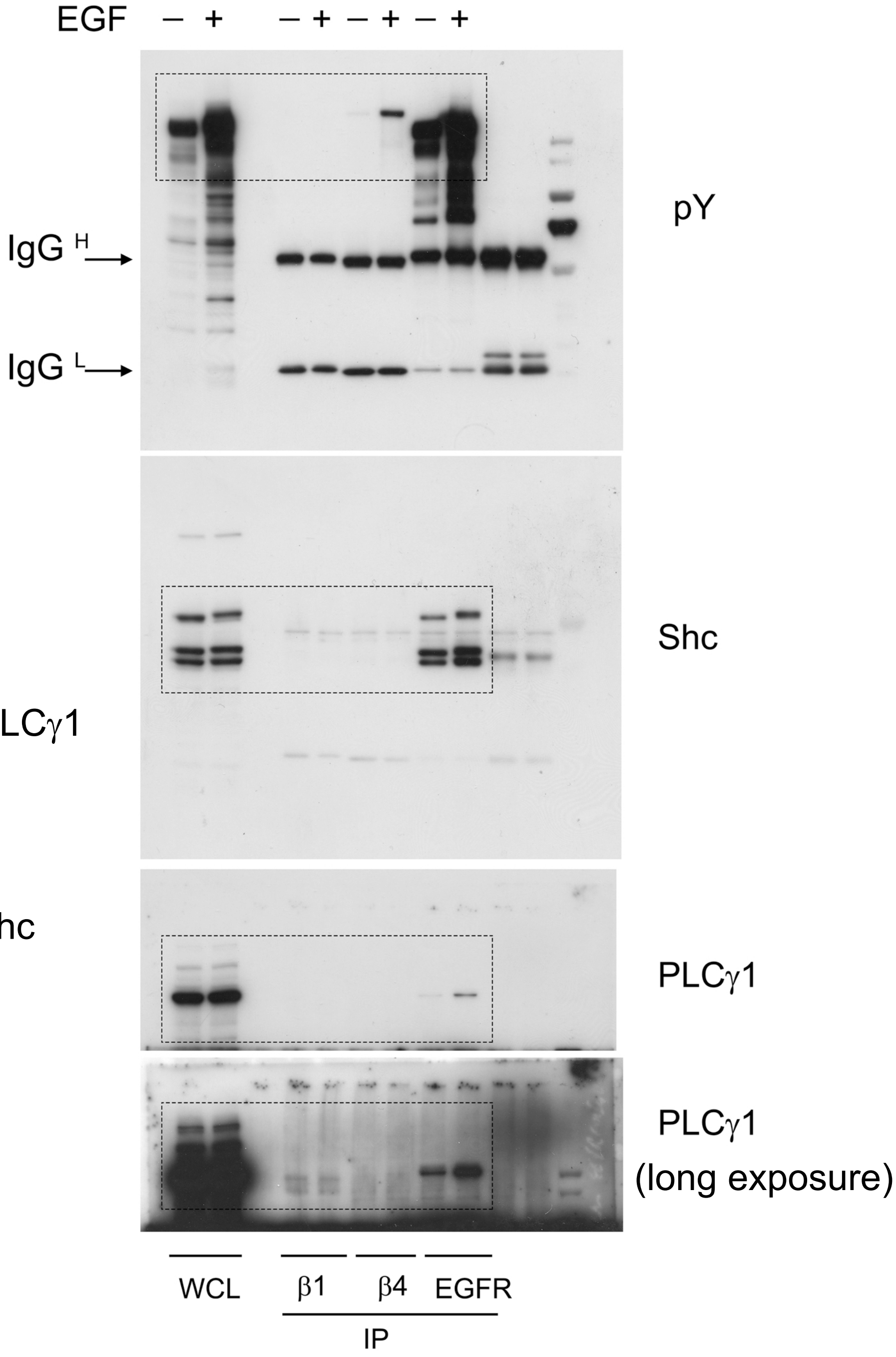

Fig. 4C

Suppl. Fig. 5: Uncropped images of Western blots in Fig. 5A, B, C and D

Fig. 5A

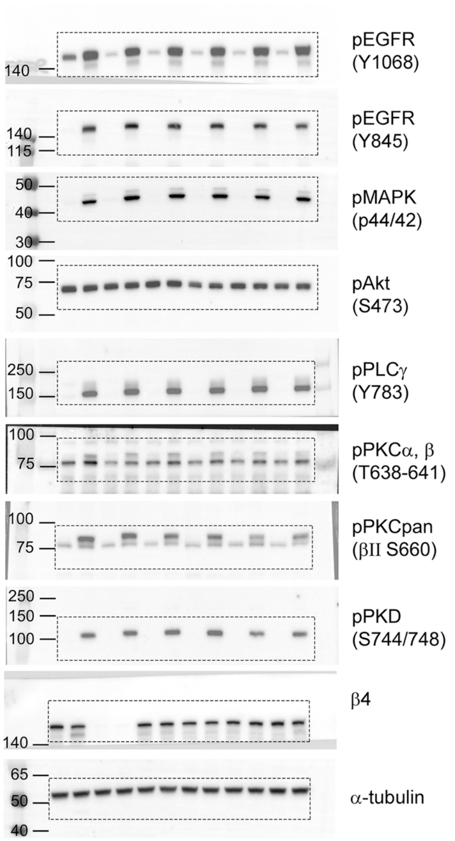

Fig. 5C

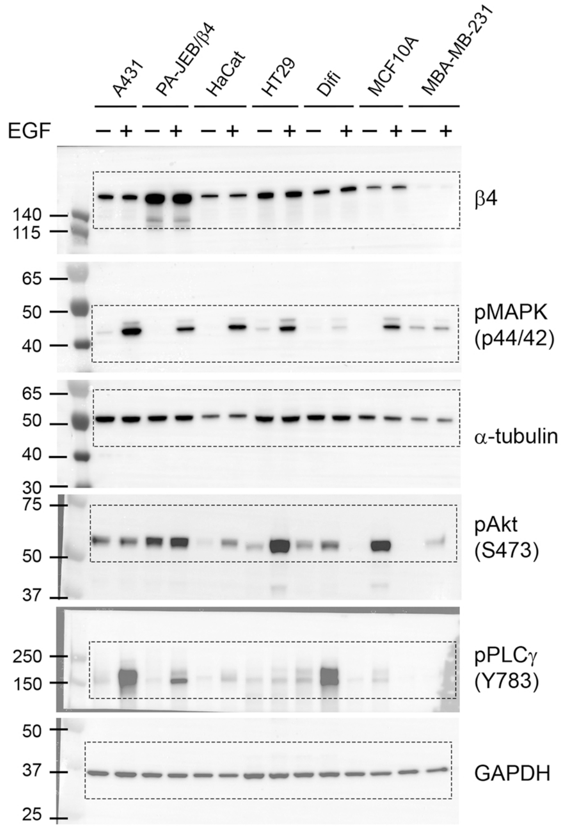

Fig. 5B

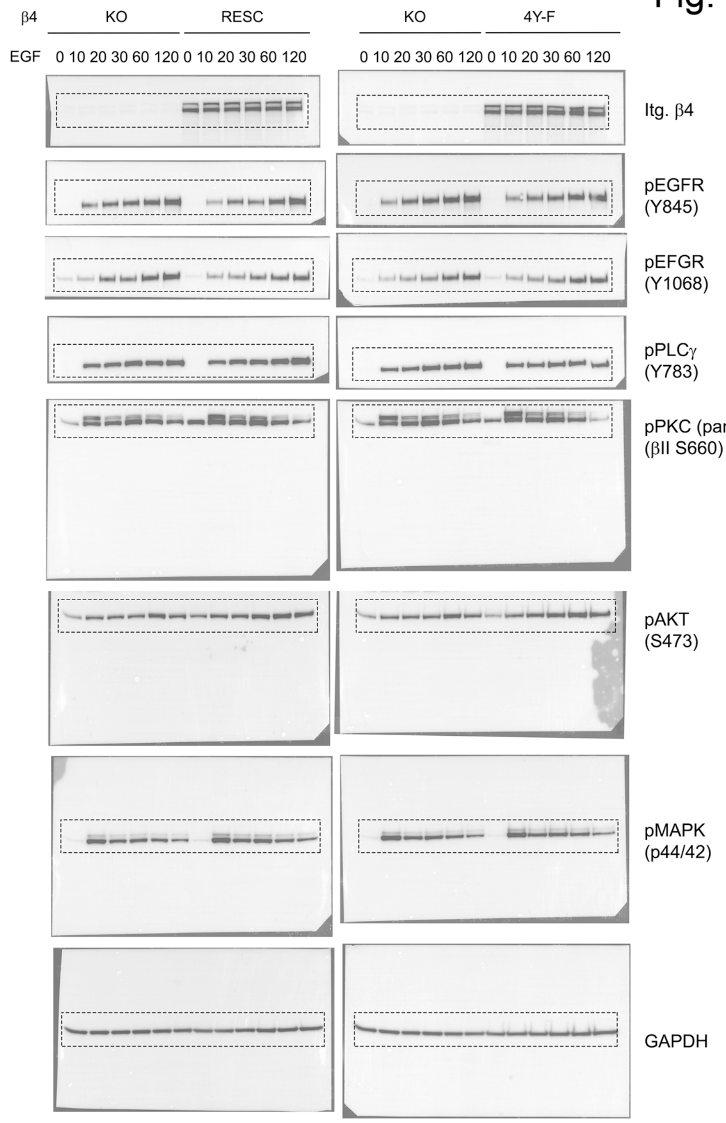

Fig. 5D

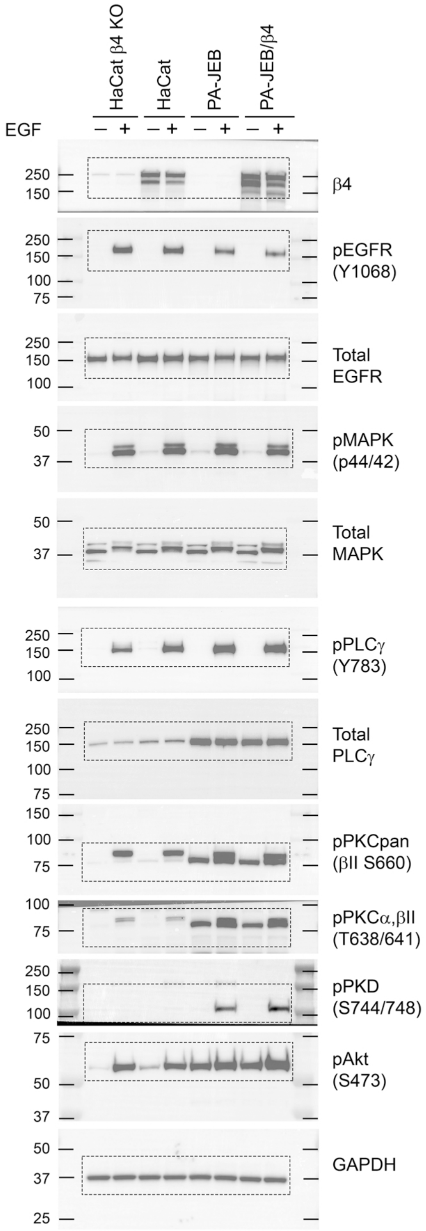

Suppl. Fig. 6: Uncropped images of merged chemiluminescent and colorimetric blots in Fig. 6A and D

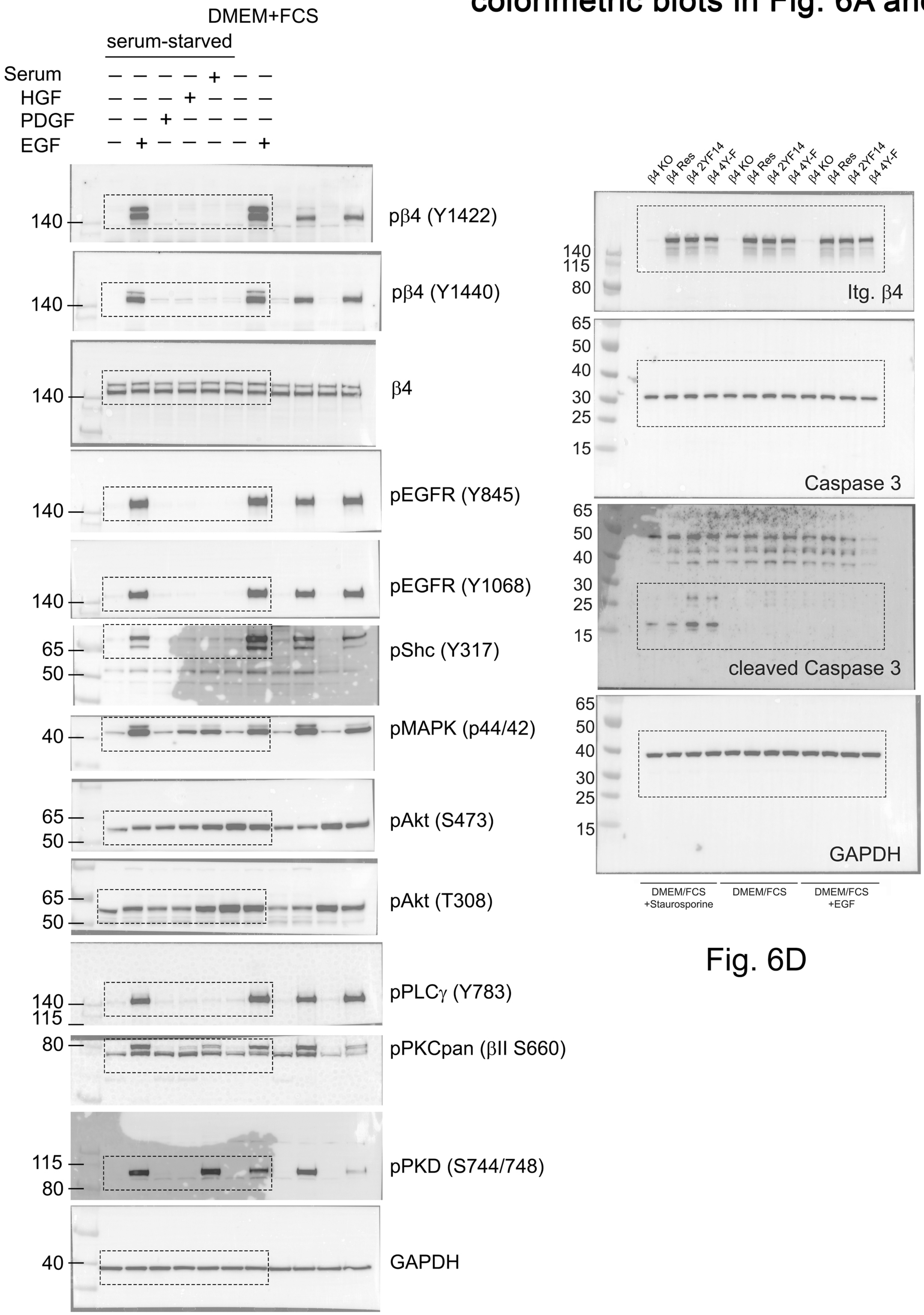

Fig. 6A

Fig. 6D
